# *Wwox* deficiency leads to neurodevelopmental and degenerative neuropathies and glycogen synthase kinase 3β-mediated epileptic seizure activity in mice

**DOI:** 10.1186/s40478-020-0883-3

**Published:** 2020-01-30

**Authors:** Ya-Yun Cheng, Ying-Tsen Chou, Feng-Jie Lai, Ming-Shiou Jan, Tsung-Hao Chang, I-Ming Jou, Pei-Shiuan Chen, Jui-Yen Lo, Shiang-Suo Huang, Nan-Shan Chang, Yung-Tsai Liou, Po-Chih Hsu, Hui-Ching Cheng, Yee-Shin Lin, Li-Jin Hsu

**Affiliations:** 1 Department of Medical Laboratory Science and Biotechnology, College of Medicine, National Cheng Kung University, 1 University Road, Tainan, 70101 Taiwan; 2 Institute of Basic Medical Sciences, College of Medicine, National Cheng Kung University, Tainan, Taiwan; 3 Department of Dermatology, Chi Mei Medical Center, Tainan, Taiwan; 4 Center for General Education, Southern Taiwan University of Science and Technology, Tainan, Taiwan; 5 Institute of Microbiology and Immunology, Chung Shan Medical University, Taichung, Taiwan; 6 Department of Orthopaedics, College of Medicine, National Cheng Kung University, Tainan, Taiwan; 7 Department of Pharmacology, Chung Shan Medical University, Taichung, Taiwan; 8 Institute of Molecular Medicine, College of Medicine, National Cheng Kung University, Tainan, Taiwan; 9 Center of Infectious Disease and Signaling Research, College of Medicine, National Cheng Kung University, Tainan, Taiwan; 10 Department of Neuroscience and Physiology, SUNY Upstate Medical University, Syracuse, New York, USA; 11 Department of Microbiology and Immunology, College of Medicine, National Cheng Kung University, Tainan, Taiwan; 12 Research Center for Medical Laboratory Biotechnology, College of Medicine, National Cheng Kung University, Tainan, Taiwan

**Keywords:** Common chromosomal fragile site, Brain malformations, Neuronal degeneration, Schwann cell apoptosis, Epilepsy

## Abstract

Human *WWOX* gene resides in the chromosomal common fragile site *FRA16D* and encodes a tumor suppressor WW domain-containing oxidoreductase. Loss-of-function mutations in both alleles of *WWOX* gene lead to autosomal recessive abnormalities in pediatric patients from consanguineous families, including microcephaly, cerebellar ataxia with epilepsy, mental retardation, retinal degeneration, developmental delay and early death. Here, we report that targeted disruption of *Wwox* gene in mice causes neurodevelopmental disorders, encompassing abnormal neuronal differentiation and migration in the brain. Cerebral malformations, such as microcephaly and incomplete separation of the hemispheres by a partial interhemispheric fissure, neuronal disorganization and heterotopia, and defective cerebellar midline fusion are observed in *Wwox*^−/−^ mice. Degenerative alterations including severe hypomyelination in the central nervous system, optic nerve atrophy, Purkinje cell loss and granular cell apoptosis in the cerebellum, and peripheral nerve demyelination due to Schwann cell apoptosis correspond to reduced amplitudes and a latency prolongation of transcranial motor evoked potentials, motor deficits and gait ataxia in *Wwox*^−/−^ mice. *Wwox* gene ablation leads to the occurrence of spontaneous epilepsy and increased susceptibility to pilocarpine- and pentylenetetrazol (PTZ)-induced seizures in preweaning mice. We determined that a significantly increased activation of glycogen synthase kinase 3β (GSK3β) occurs in *Wwox*^−/−^ mouse cerebral cortex, hippocampus and cerebellum. Inhibition of GSK3β by lithium ion significantly abolishes the onset of PTZ-induced seizure in *Wwox*^−/−^ mice. Together, our findings reveal that the neurodevelopmental and neurodegenerative deficits in *Wwox* knockout mice strikingly recapitulate the key features of human neuropathies, and that targeting GSK3β with lithium ion ameliorates epilepsy.

## Introduction

Common fragile sites are large chromosomal regions that tend to form gaps or breaks under replication stress. Genomic instability and alterations at the chromosomal fragile sites have been implicated as being causative for many types of human cancers [23]. Interestingly, mutations in the genes residing within the common fragile regions, such as the genes encoding PARKIN, GRID2, CNTNAP2, Disabled-1 and LRP1B, have been shown to be associated with neurological disorders, including juvenile parkinsonism, cerebellar ataxia and atrophy, neuronal migration abnormalities during development, epileptic seizures, autism and Alzheimer’s disease [14, 26, 27, 32, 51, 54, 63, 64]. How genomic alterations at common fragile sites lead to neuropathology is largely unclear.

Human *WWOX* gene is mapped to a common fragile site *FRA16D* on chromosome 16q23.3–24.1, and encodes a tumor suppressor WW domain-containing oxidoreductase, WWOX [11, 17, 56]. Deletions, loss of heterozygosity and translocations of *WWOX* gene have been frequently observed in various human malignancies, such as breast, prostate, ovarian, esophageal, lung, stomach, and pancreatic cancers [16, 44]. Downregulation of proapoptotic WWOX expression is associated with cancer progression [7, 37]. Recent studies have suggested that WWOX may act more than a tumor suppressor. Upon neuronal injury, WWOX is activated via phosphorylation at tyrosine 33 and translocates to the mitochondria and nucleus [18, 41]. In a rat model of Parkinson’s disease, treatment of 1-methyl-4-phenyl-pyridinium (MPP^+^) rapidly increases complex formation of WWOX and JNK1, followed by nuclear accumulation of WWOX and neuronal death in the cortical and striatal neurons [43]. WWOX protein expression is significantly downregulated in the hippocampal neurons of patients with Alzheimer’s disease [59]. Suppression of WWOX expression by small interfering RNA induces Tau hyperphosphorylation and formation of neurofibrillary tangles in neuroblastoma SK-N-SH cells, suggesting a crucial role of WWOX in inhibiting Tau phosphorylation in the degenerative neurons of Alzheimer’s disease [15, 58, 59]. *Wwox*-deficient mice are significantly reduced in size, exhibit abnormalities of bone metabolism and succumb to death by 4 weeks postnatally [8, 9]. In additional to the inhibition of runt-related transcription factor 2 for regulating osteoblast differentiation and bone tissue formation, WWOX also suppresses the transactivation ability of hypoxia-inducible transcription factor 1α for controlling glucose metabolism and mitochondrial respiration [3, 8]. Although WWOX has been demonstrated to exert its functions through regulating many signaling molecules, the vital requirements for WWOX in vivo remain largely undefined.

During mouse embryonic development, WWOX is highly expressed in the neural crest-derived structures such as cranial and spinal ganglia, skin pigment cells and mesenchyme in the head, suggesting possible involvement of WWOX in neuronal differentiation and maturation [19]. WWOX has been shown to interact with and inhibit glycogen synthase kinase 3β (GSK3β) for promoting microtubule assembly activity of Tau and neurite outgrowth during retinoic acid-induced SH-SY5Y neuronal differentiation [65]. Of note, similar to a spontaneous *lde* mutant rat model, the phenotypes of patients with homozygous loss-of-function mutations of *WWOX* gene from consanguineous families include microcephaly, cerebellar ataxia associated with epileptic seizures and mental retardation, retinopathy, profound developmental delay, and premature death [2, 12, 22, 35, 48, 50, 57, 60, 61]. However, the neurodevelopmental deficits due to functional loss of WWOX remain undefined. In the developing brain, immature neurons migrate outwards from the neuroectoderm to their defined locations, giving rise to characteristic cell layers. Here, we show that targeted disruption of *Wwox* gene in mice disturbs neuronal migration in the cerebral cortex, hippocampus and cerebellum. Remarkably, our generated *Wwox* knockout mice recapitulate the key features of human neuropathies, including brain malformations and neuronal degeneration along with epilepsy and motor disorders, making them a valuable disease model in which to delineate the developmental and pathological processes that lead to central and peripheral nerve dysfunction.

## Materials and methods

### *Wwox* gene knockout mice, rotarod performance and footprint analysis

Mouse *Wwox* gene locates on chromosome band 8E1 and consists of nine exons, giving rise to a ~ 2.2 kb transcript. The exon 1 of *Wwox* contains the 5′-UTR and a start codon for translation of a 46-kDa full-length protein. A previous study has developed a *Wwox* knockout mouse model by targeting exons 2/3/4 [9]. To test if the possibly generated aberrant protein may cause phenotypes due to the presence of exon 1 in the mouse genome, we generated both exon 1- and exon 2/3/4-targeting knockout mouse strains for comparison (Additional file 1, online resource). Mice were maintained on standard laboratory chow and water ad libitum in a specific pathogen-free environment. The experimental procedures were carried out in strict accordance with approved protocols for animal use from the Institutional Animal Care and Use Committee of National Cheng Kung University.

The tests for motor coordination and balance were performed in mice at 18–20 days of age according to the procedures described previously [13]. For rotarod tests, mice were acclimatized to a rotarod (Ugo Basile Model 7650-RotaRod Treadmill) rotating at 5 rpm for 5 min, and a 10-min intertrial interval was allowed in the training period. Four trials per day for three consecutive days were conducted prior to data acquisition. For the constant speed rotarod test, each mouse was placed individually on the rotating rod set at a fixed speed, and the latency to fall off the rotating rod was measured. For the accelerating rotarod test, the assessment began at 4 rpm and gradually increased to a maximum speed of 40 rpm over a period of 5 min. If the mouse stayed on the rod till the end of 10-min trial, a time of 600 s was recorded. Mice were given two trials each day for five consecutive days. The mean values were used for statistical comparison.

For footprint analysis, mouse forepaws were dipped in nontoxic water-based red ink, and hind paws in blue. The mice were then allowed to walk along an enclosed runway and leave a set of footprints on white paper. The stride length, base width and the hind/fore-base ratio were measured for mouse gait analysis. At least five steps were measured for each mouse, and the mean of values was used for analysis.

### Recording of transcranial motor evoked potentials (Tc-MEPs)

Mice were intraperitoneally anesthetized with chloral hydrate in PBS (400 mg/kg; Tokyo Chemical Industry, product no. C0073). The depth of anesthesia was monitored by withdrawal reflex upon tail pinch. Core temperature was monitored using a rectal probe connected to a multichannel thermometer (Portable Hybrid Recorder, model 3087; Yokogawa Hokushin Electric, Tokyo, Japan) and maintained at 37 °C by heating pads and lamp. Tc-MEPs were recorded using monopolar myographic needle electrodes placed in the intrinsic plantar muscles of bilateral forelimbs. A ground electrode was placed subcutaneously between the stimulating and the recording sites. The stimulus was applied with a duration of 0.2 msec in a series of square pulses using two needle electrodes attached to the scalp. The presentation rate of stimulation was 1/s. The supramaximal stimulus was assessed and the recording was performed at an intensity of 10% above the stimulus level that produced maximal amplitudes. The recording time was 10 msec, and the recorded signals were amplified and filtered between 1 and 2000 Hz. At least three sequential single-sweep runs (i.e.*,* without averaging) with similar waveforms were recorded to verify the consistency of responses. The electrophysiological data were collected, processed and analyzed on a Neuropack Z recording device (Nihon Koden, Tokyo, Japan). Amplitude of the Tc-MEPs was defined as the peak-to-peak distance in microvolts (μV), and latency of the response was measured from the onset of electrical shock artifact to the major positive peak in msec.

### Immunoelectron microscopy, luxol fast blue (LFB) and cresyl violet staining, immunohistochemistry and terminal deoxynucleotidyl transferase dUTP nick end labeling (TUNEL) assay

Mouse sciatic nerves were fixed in 4% glutaraldehyde/0.1 M cacodylate buffer (pH 7.2), dehydrated and embedded in EMbed 812 resin (Electron Microscopy Sciences) in a 60 °C oven. Semi-thin transverse sciatic nerve sections (800 nm; Leica EM UC6 ultramicrotome) were stained with 1% toluidine blue/1% azure/1% sodium borate in H_2_O for 30 s, and examined under a light microscopy (Olympus BX51). For electron microscopy, ultrathin sections (90 nm) were prepared, incubated with rabbit anti-cleaved caspase-3 (Asp175) antibody (Cell Signaling), and then stained with 20-nm gold particle-conjugated anti-rabbit IgG (BB International Ltd). The samples were further stained with 2% uranyl acetate for 20 min and 4% lead citrate for 3 min, and examined under a transmission electron microscopy (Hitachi H-7000).

Mouse brains or embryos were fixed in 3.7% formaldehyde in PBS and embedded in paraffin. Five-μm tissue sections on glass slides were deparaffinized, hydrated through serial concentrations of ethanol and finally in distilled water. The brain sections were incubated in 0.1% LFB/0.5% acetic acid/95% ethanol solution at 56 °C overnight, rinsed in 95% ethanol and then distilled water, and differentiated in 0.05% lithium carbonate solution for 30 s. The samples were counterstained in 0.1% cresyl violet solution for 6 min, dehydrated in 95% and absolute ethanol, cleared in xylene, and mounted. Immunohistochemistry of the 5-μm tissue sections was performed as described previously [37], using specific antibodies against doublecortin (DCX) (1:40, Santa Cruz and GeneTex), NeuN (1:2000 dilution, Millipore), calbindin (1:500, Sigma) and Ki67 (1:150, Dako) in Dako diluent. After incubating with a secondary antibody and NovoLink polymer (Leica Biosystems), the tissue sections were treated with 3-amino-9-ethylcarbazole (AEC) substrate chromogen (Zymed), counterstained with hematoxylin solution, and mounted in aqueous mounting media.

For TUNEL assay, an ApopTag plus peroxidase in situ apoptosis detection kit (Millipore) was used to analyze DNA fragmentation in cells according to the manufacturer’s protocol. In brief, deparaffinized brain sections were rehydrated, incubated with proteinase K (20 μg/ml) at room temperature for 15 min, and treated with 3% hydrogen peroxide in PBS for 10 min to quench the endogenous peroxidase activity. After equilibration, the samples were incubated with terminal deoxynucleotidyl transferase in reaction buffer containing digoxigenin-conjugated nucleotides at 37 °C for 1 h to label the free DNA termini. The incorporated nucleotides within the fragmented DNA were detected by the binding of peroxdase-conjugated anti-digoxigenin antibody, followed by the addition of AEC substrate chromogen. The tissue sections were counterstained with hematoxylin solution for 10 min at room temperature, and TUNEL-positive cells were visualized under an Olympus BX51 microscope.

### Western blotting

Cerebellum, hippocampus and cerebral cortex tissues were isolated from three genotypes of mice at postnatal day 14 for protein extraction using a lysis buffer containing 0.1% SDS, 1% Nonidet P-40, 0.5% Tween 20, 10 mM Na_4_P_2_O_7_, 10 mM Na_3_VO_4_, 10 mM NaF, and 1:20 dilution of protease inhibitor cocktail (Sigma) in PBS. Western blot analysis was performed as described previously [62], using anti-WWOX, anti-DCX (GeneTex), and anti-β-actin (Sigma) antibodies.

### Induction of seizure

Methylscopolamine bromide, pilocarpine, pentylenetetrazol (PTZ), ethosuximide and lithium chloride (LiCl) were purchased from Sigma-Aldrich, and dissolved in 0.9% sodium chloride freshly before use. For pilocarpine-induced seizure model, the mice were intraperitoneally (i.p.) pretreated with methylscopolamine bromide (1 mg/kg) 30 min prior to pilocarpine administration to limit peripheral cholinergic effects, and then injected with pilocarpine (i.p., 50 mg/kg). After methylscopolamine pretreatment, the control mice were given an equal volume of saline. For PTZ model, we injected PTZ i.p. to mice at a dose of 30 mg/kg [46]. Following pilocarpine or PTZ injection into the mice, the seizure severity was assessed for 60 min according to a modified version of Racine scale: stage 0, no response; stage 1, behavioral arrest followed by vibrissae twitching; stage 2, head nodding; stage 3, unilateral forelimb clonus and myoclonic jerk; stage 4, bilateral forelimb clonus with rearing; stage 5, generalized tonic-clonic seizure (GTCS) and loss of righting reflex; stage 6, dead [55]. Ethosuximide (i.p., 150 mg/kg), a T-type Ca^2+^ channel blocker that has anticonvulsant activity (Luszczki et al., 2005), was injected into mice 45 min before PTZ-induced clonic seizures. LiCl (i.p., 60 mg/kg) were pretreated three times within 1 h before PTZ injection.

### Statistical analysis

We performed statistical tests with one-way analysis of variance (ANOVA) to compare the difference among groups. The differences were considered significant when the *P* values were less than 0.05. All results are presented as means ± standard error of the mean (SEM).

## Results

### Neurological motor disorders in *Wwox* gene knockout mice

We have developed two knockout mouse models with ablation of exon 1 or exons 2/3/4 of *Wwox* gene (WD1 or WD234 henceforth, respectively). Southern blot analysis using genomic DNA isolated from mouse embryonic fibroblasts (MEF) and polymerase chain reactions using mouse tail DNA demonstrated that *Wwox* gene was disrupted in both WD1 and WD234 mice (Additional file 1: Figure S1a, b). There was undetectable protein expression in the homozygous *Wwox* knockout (*Wwox*^−/−^) MEF (Additional file 1: Figure S1c). In agreement with a previous study [8], our generated *Wwox*^−/−^ mice with exon 1- or exon 2/3/4-deletion showed severe dwarfism and survived for less than a month (Additional file 1: Figure S1d).

**Fig. 1:**
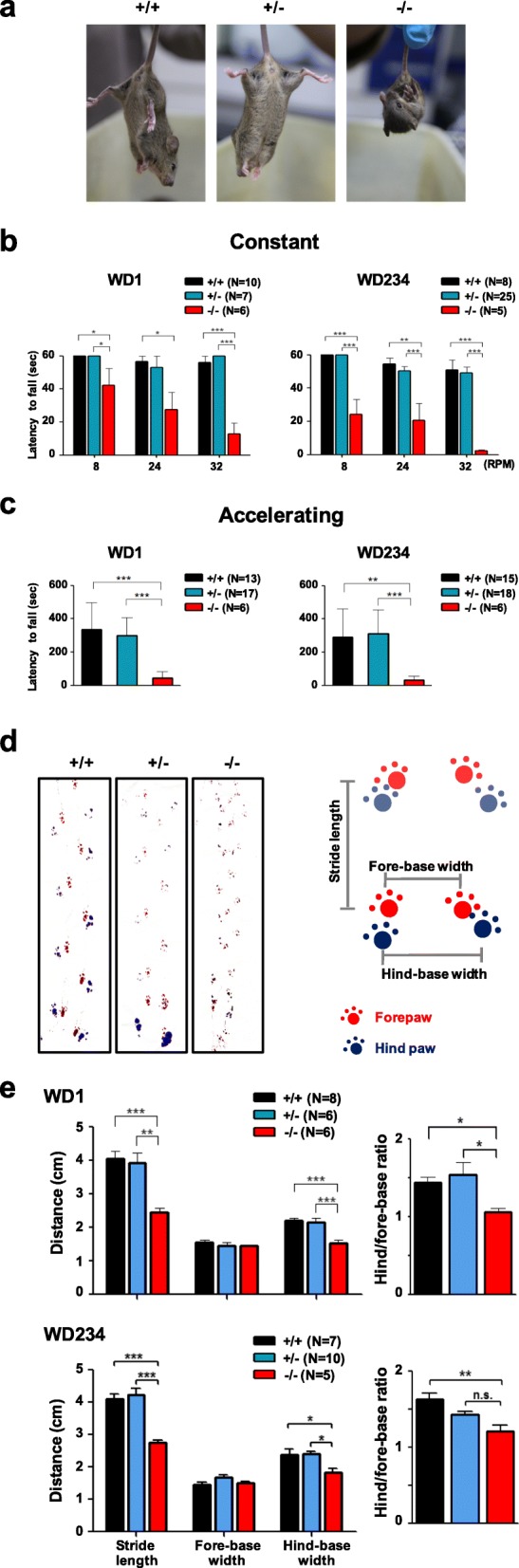
*Wwox*^*−/−*^ mice exhibit motor disorders. **a** Tail suspension test revealed abnormal limb-clasping reflex in *Wwox*^*−/−*^ mice at postnatal day 20. **b**, **c** Rotarod analysis of motor function in three genotypes of both WD1 and WD234 mice was performed on a constant-speed (**b**) or accelerating rotarod (**c**). The latencies from rotation onset until the mice fell off the rod were recorded. *Wwox*^*+/+*^ and *Wwox*^*+/−*^ mice managed to stay significantly longer on the rotarod than *Wwox*^*−/−*^ mice. **d**, **e** Footprint analysis of gait abnormalities in *Wwox*^*−/−*^ mice. Mouse forepaws were marked with red ink and hind paws with blue for gait assessment. The mice with ink on their paws were trained to run down a corridor and the mouse gait patters of three genotypes were obtained (**d**). The stride length and hind-base width of *Wwox*^*−/−*^ mice were significantly shorter than those of *Wwox*^*+/+*^ and *Wwox*^*+/−*^ mice (**e**). Also, the hind to fore-base ratios were lower in *Wwox*^*−/−*^ mice compared with their littermates (**e**). Differences between *Wwox*^*−/−*^ versus *Wwox*^*+/+*^ and *Wwox*^*+/−*^ littermates were statistically significant in a one-way ANOVA test. Each result represents the average of data obtained and error bars are standard error of the mean (SEM). n.s., non-significant. *P < 0.05, **P < 0.01, ***P < 0.001; RPM, revolutions per minute; N, number of animals tested

High expression levels of WWOX protein have been observed in the neural crest-derived structures such as cranial and spinal ganglia, skin pigment cells and mesenchyme in the head of mouse embryo, suggesting possible involvement of WWOX in neuronal differentiation [19]. Compared with the *Wwox*^+/+^ and *Wwox*^+/−^ littermates, reduction in brain size and weight was observed in *Wwox*^−/−^ mice at postnatal day 20 (Additional file 1: Figure S1e and S1f for WD1, respectively, and data not shown for WD234). As in *Wwox* knockout mouse models, a homozygous *WWOX* nonsense mutation caused growth retardation, microcephaly and early death in a patient from a consanguineous family [2]. No differences in brain water contents were detected among the mice of three genotypes (Additional file 1: Figure S1 g). To assess the role of WWOX in neuronal functions, *Wwox*^−/−^ mice were first examined for their motor coordination phenotypes. *Wwox*^*+/+*^ and *Wwox*^*+/−*^ mice exhibited a normal plantar reaction when they were suspended by their tails, whereas *Wwox*^−/−^ mice showed abnormal hind limb-clasping reflexes (Fig. 1a). In rotarod tests, *Wwox*^−/−^ mice had much shorter latency periods before falling off the rotating rotarod at either constant or accelerating speeds than their *Wwox*^*+/+*^ and *Wwox*^*+/−*^ littermates (Fig. 1b, c). Moreover, a footprint assay was performed to record gait abnormalities in *Wwox* deficient mice. *Wwox*^−/−^ mice showed uncoordinated movements and overlapped footprints of their fore and hind paws (Fig. 1d). Our data showed that the stride length, hind-base width, and hind-to-fore base ratio were significantly decreased in *Wwox*^−/−^ mice (Fig. 1d, e). Similar results were obtained when analyzing the ratio of stride length or hind-base width to the body size (data not shown). There were no significant differences between *Wwox*^*+/+*^ and *Wwox*^*+/−*^ mice (Fig. 1b-e). We performed rotarod tests and footprint assay with both WD1 and WD234 mice and obtained similar results (Fig. 1b-e). Our results indicate that *Wwox* gene ablation in mice leads to gait ataxia and severe impairment in their motor coordination, grip strength and balance.

### Nerve degeneration and demyelination in *Wwox*^−/−^ mice

**Fig. 2:**
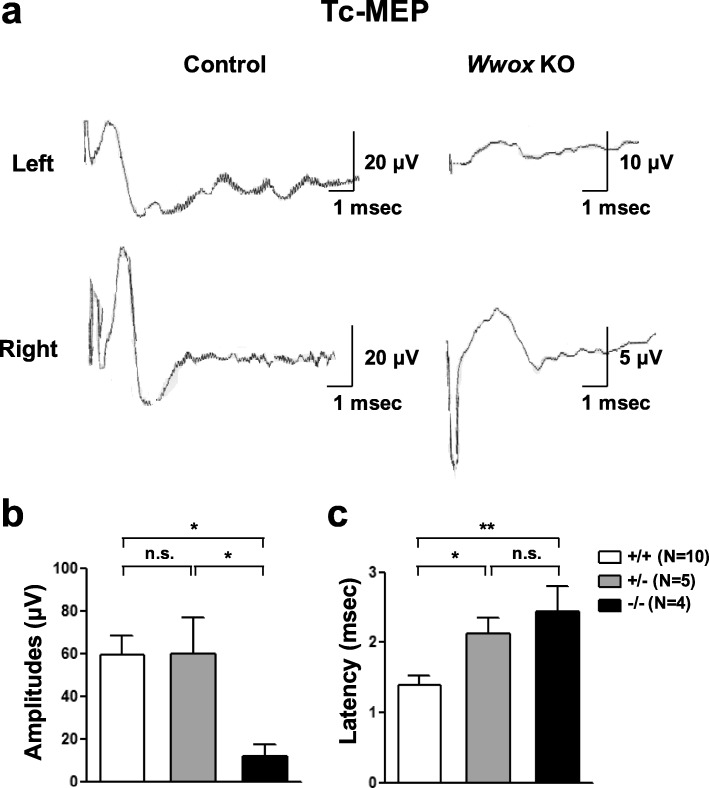
*Wwox* knockout in mice leads to the changes in Tc-MEPs. **a** Representative bilateral Tc-MEPs detected in wild-type control and *Wwox* knockout mice. When compared to *Wwox*^*+/+*^ mice, significantly reduced amplitudes (**b**) and increased latencies of Tc-MEPs (**c**) were determined in *Wwox*^−/−^ mice at 3 weeks of age. A statistically significant increase in the Tc-MEP latency, but no changes in amplitude, was observed in *Wwox*^+/−^ mice, indicating that haploinsufficiency of *Wwox* gene may cause a delay in wave latencies with no effect on their amplitudes. The results are expressed as means ± SEM. n.s., non-significant. *P < 0.05, **P < 0.01

Motor neuropathies may lead to defects in movement coordination. To understand whether *Wwox* deficit leads to abnormalities in the functional state of motor nervous system, Tc-MEPs were recorded in three genotypes of mice at 3 weeks of age. The Tc-MEPs elicited by electrical stimulation of the motor cortex monitor the descending response that is propagated through the corticospinal tracts to cause a muscle contraction. Compared with the results recorded in *Wwox*^+/+^ mice (59.2 ± 9.0 μV; *n* = 10), a significant reduction in the amplitudes of Tc-MEPs with an average of 11.8 ± 5.4 μV was analyzed in *Wwox*^−/−^ mice (Fig. 2a, b; *n* = 4, *p* < 0.05). The onset latency of Tc-MEP showed a significant prolongation in *Wwox*^−/−^ (2.44 ± 0.37 msec) than *Wwox*^+/+^ mice (1.39 ± 0.13 msec) (Fig. 2c; *p* < 0.01). Although the mean amplitude of Tc-MEP recoded in *Wwox*^+/−^ mice (59.6 ± 17.2 μV; *n* = 5) was comparable to *Wwox*^+/+^ mice, an increase in Tc-MEP latency was determined in *Wwox*^+/−^ mice (2.13 ± 0.22 msec; *p* < 0.05) when compared with the wild-type mice (Fig. 2b, c), suggesting that *Wwox* haploinsufficiency may cause a partial Tc-MEP deteriorative change in mice.

**Fig. 3:**
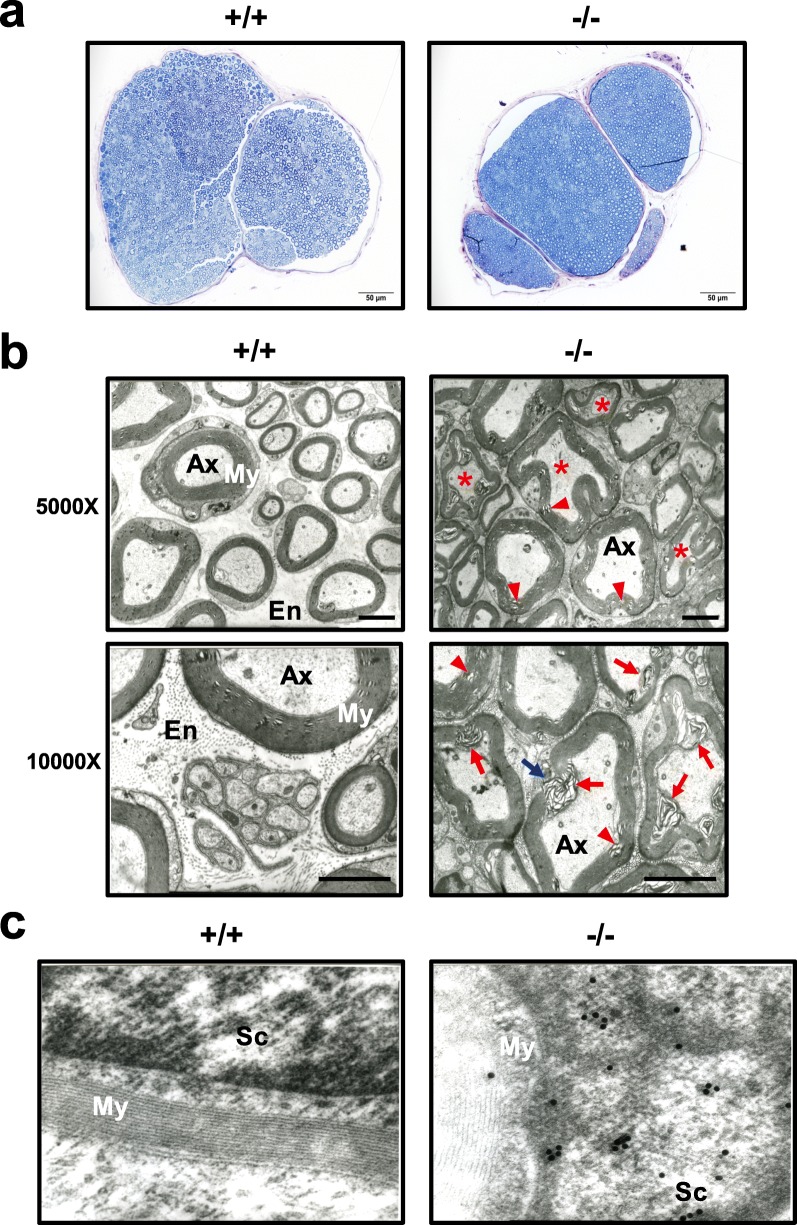
Peripheral nerve degeneration and Schwann cell apoptosis in *Wwox*^−/−^ mice. **a** Semi-thin toluidine blue-stained transverse sections of sciatic nerves embedded in EMbed 812 resin from *Wwox*^*+/+*^ and *Wwox*^*−/−*^ mice at postnatal day 20 are shown (*N* = 3). Scale bars = 50 μm. **b** Electron microscopy revealed normal ultrastructural features of axons (Ax), myelin sheath (My) and endoneurium (En) in EMbed 812 resin-embedded sciatic nerve sections from *Wwox*^*+/+*^ mice. In contrast, abnormal-shaped nerve fibers (red stars), axon demyelination (blue arrow) and onion bulb degeneration (red arrowheads) were observed in all *Wwox*^*−/−*^ sciatic nerves examined in this study (N = 3). Red arrows indicate detachment of myelin lamellae with invasion towards the axolemma due to loss of axoplasm in *Wwox*^*−/−*^ axons. A significant reduction in endoneurium spaces was evident in *Wwox*^*−/−*^ sciatic nerves. Scale bars = 5 μm. **c** By immunogold-labelling, cleaved caspase-3 was detected in the Schwann cell (Sc) from *Wwox*^*−/−*^ sciatic nerve, photographed at 50,000X magnification. The representative images of three independent experiments are shown

We then assessed whether the alterations in neurophysiological function were supported by the presence of neuropathological changes in *Wwox*^−/−^ mice. Semi-thin transverse sciatic nerve sections stained with toluidine blue revealed similar axonal organization and approximately equal numbers of nerve fibers in wild-type and *Wwox*^−/−^ mice (Fig. 3a). However, smaller endoneurium spaces were observed in *Wwox*^−/−^ mouse sciatic nerves (Fig. 3a, b). Strikingly, a large number of abnormal-shaped and demyelinated axons in a compact mass were found in the sciatic nerves of *Wwox*^−/−^ mice by transmission electron microscopy (Fig. 3b). Detachment of myelin lamellae and loss of axoplasm were evident in the degenerative sciatic nerve fibers of *Wwox*^−/−^ mice (Fig. 3b). Myelin thickness, axonal organization and the density of myelinated fibers were similar in *Wwox*^+/−^ and *Wwox*^+/+^ mice (data not shown). Schwann cells produce the myelin sheath around axons in the peripheral nervous system (PNS). A cleaved form of active caspase-3 was detected in the Schwann cells of *Wwox*^−/−^ mice by immunoelectron microscopy (Fig. 3c), indicating that *Wwox* deficit may cause Schwann cell apoptosis and axon demyelination in the PNS.

**Fig. 4:**
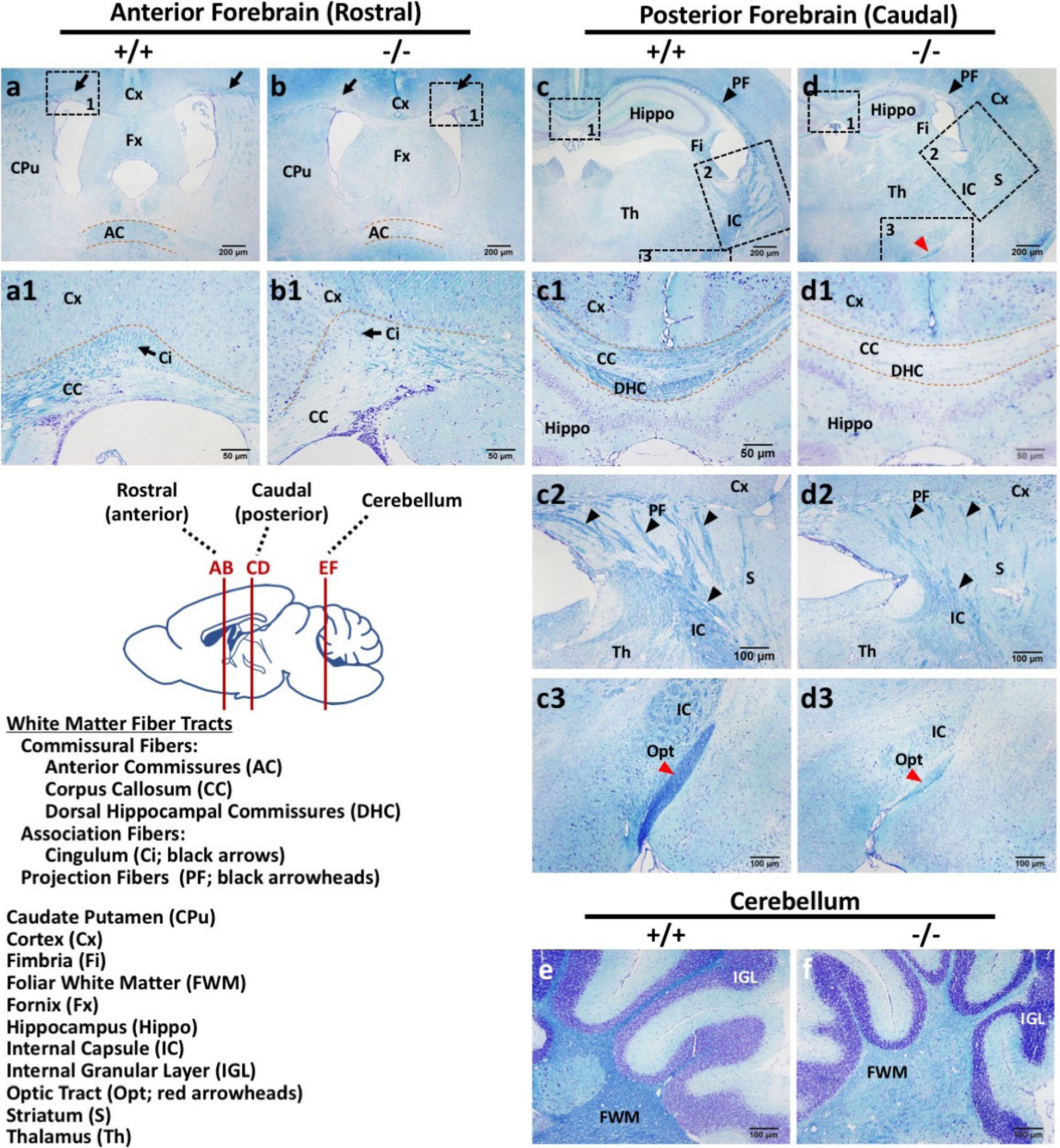
*Wwox* loss results in severe CNS hypomyelination in mice. **a**-**d** LFB staining of CNS white matter fiber tracks using mouse forebrain coronal sections showed that the myelinated neurons were largely reduced in the commissural fibers (corpus callosum, anterior commissures and dorsal hippocampal commissures), association fibers (cingulum; black arrows) and projection fibers (black arrowheads) of all *Wwox*^*−/−*^ mice examined at 3 week of age. The enlarged images (**a1**, **b1**, **c1–3**, and **d1–3**) are from the boxed areas in the upper panel (**a**-**d**). Myelin pallor was also observed in the internal capsule (**c** and **d**), degenerated optic tract (**c3** and **d3**; red arrowheads) and cerebellar foliar white matter (**e** and **f**) of *Wwox*^*−/−*^ mice by LFB staining. Nissl staining of neuronal cell bodies was used for counterstaining. The representative results of three independent experiments are shown

Brain magnetic resonance imaging has revealed poor myelination and progressive atrophy of periventricular white matter resulting in hypoplastic corpus callosum in patients with homozygous mutation in *WWOX* gene [2, 22, 50, 60]. LFB staining of myelin was performed to examine the white matter fiber tracts in *Wwox*^−/−^ mouse brain. Compared to the wild-type with regular myelination, *Wwox*^−/−^ mouse brain sections showed significantly reduced myelin staining intensity in the commissural fibers (corpus callosum, and anterior and dorsal hippocampal commissures), the association fibers (cingulum), and the projection fibers emanating from corpus callosum toward striatum (Fig. 4a-d). The commissural fibers communicate between two cerebral hemispheres and the association fibers connect regions within the same hemisphere of the brain. Myelin pallor was also observed in *Wwox*^*−/−*^ internal capsule, where both ascending and descending axons going to and coming from the cerebral cortex pass through (Fig. 4c, d). Of note, hypomyelination with atrophy of optic tract and cerebellar foliar white matter was examined in *Wwox*^*−/−*^ mice (Fig. 4c3, d3, e, f). Together, our results provide clear neuropathological findings indicating that severe hypomyelination in the PNS and brain of *Wwox*^−/−^ mice.

### Cerebellar hypoplasia with a defective vermis-midline fusion in *Wwox*^−/−^ mice

**Fig. 5:**
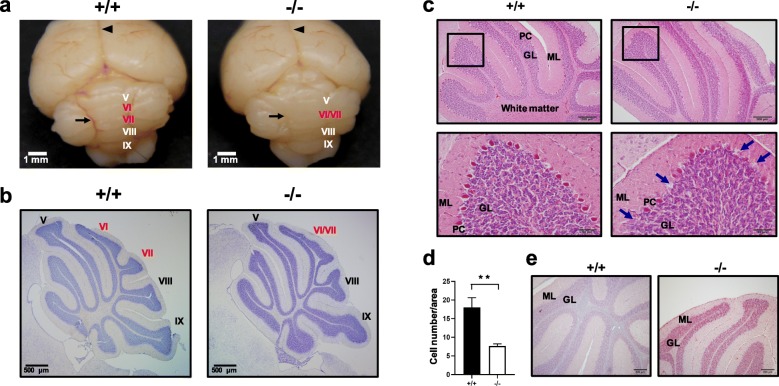
Foliation defect, Purkinje cell loss and neuronal apoptosis in the cerebellum of *Wwox*^*−/−*^ mice*.* **a** Representative images of *Wwox*^*+/+*^ and *Wwox*^*−/−*^ brains revealed that interhemispheric fissure (arrowheads) and cerebellar vermian lobules VI and VII (arrow) were fused in *Wwox*^*−/−*^ brain. **b** Midline sagittal sections of *Wwox*^*+/+*^ and *Wwox*^*−/−*^ cerebellum tissues were stained with cresyl violet. A smaller lobule V and fusion of VI with VII were observed in *Wwox*^*−/−*^ mouse cerebellum. Scale bars = 500 μm. **c** H&E staining of the cerebellar cortex tissue sections from *Wwox*^*−/−*^ mice (*N* = 6) showed partial loss of Purkinje cells (arrows) at postnatal day 20. The lower panel pictures are the magnified images from the boxed area in the upper panel. Scale bars = 200 (upper) or 50 μm (lower). **d** The numbers of Purkinje cells in ten representative subareas of *Wwox*^*+/+*^ and *Wwox*^*−/−*^ cerebellar cortex tissue sections were quantified. The results are expressed as means ± SEM. **P < 0.01. **e** Apoptotic cell death was detected in the granular cells of *Wwox*^*−/−*^ cerebellum at postnatal day 20 by TUNEL assay. The representative results of four independent experiments are shown. PC, Purkinje cell; GL, Granule cell layer; ML, molecular layer. Scale bars = 200 μm

Cerebellar modulation and coordination of neuromuscular activities are important in skilled voluntary movement and equilibrium. Cerebellar foliation along the anterior-posterior axis of the vermis during development contributes to increased surface area, permitting the cerebellum to accommodate more cells and facilitating the establishment of more sophisticated sensory-motor circuits. In *Wwox*^−/−^ mice, an aberrant cerebellum with fusion of vermian lobules VI and VII was observed (Fig. 5a). Histological examination was performed to evaluate developmental changes in the cerebellum of *Wwox*^−/−^ mice. Midline sagittal cresyl violet-stained section revealed foliation defects in lobules V, VI and VII of *Wwox*^−/−^ cerebellum (Fig. 5b). Technologically, fusion of vermian lobules VI and VII and a smaller lobule V were observed at postnatal day 19~20 in *Wwox*^−/−^ cerebellum (Fig. 5b). Furthermore, staining results of hematoxylin and eosin (H&E) (Fig. 5c, d) and immunofluorescence using an antibody against calbindin, a selective marker for Purkinje cells in the cerebellum (Additional file 1: Figure S2), demonstrated partial loss of the Purkinje cells and their diminished expression of calbindin in *Wwox*^−/−^ cerebellum at postnatal day 20. TUNEL assay showed increased apoptotic cells in the adjacent granular layer of *Wwox*^−/−^ cerebellum (Fig. 5e and Additional file 1: Figure S3). Together, aberrant foliation, Purkinje cell loss and neuronal apoptosis in the cerebellum may contribute to early postnatal ataxia in *Wwox*^−/−^ mice.

### *Wwox* is required for proper neuronal migration and development

The homozygous *Wwox* knockout mice were found along a spectrum of phenotypic severity, and some very severely affected embryos died embryonically. Gross morphological abnormalities could be observed in *Wwox*^−/−^ mouse brains from live births, ranging from microcephaly to holoprosencephaly, in which the forebrain did not properly divide into two hemispheres during embryonic development. Technically, middle interhemispheric fusion of the posterior frontal and parietal lobes was found in *Wwox*^−/−^ mouse brains (Fig. 5a and Additional file 1: Figure S4). Given the striking brain morphological phenotypes in *Wwox*^−/−^ mice, we investigated the function of WWOX during neural development. *Wwox*^−/−^ mouse embryos at E12.5 were smaller in size and exhibited a growth delay as compared with the wild-type littermates (Additional file 1: Figure S5a). An elongated roof plate and dorsal spinal cord malformation were evident in E12.5 *Wwox* null embryos (Additional file 1: Figure S5a). In the developing brain, Ki-67^+^ proliferating cells were decreased in *Wwox*^−/−^ neocortical subventricular zone and cerebellum compared with the wild-types at E16.5 (Additional file 1: Figure S5b). We found that the overall cortical thickness was significantly reduced in *Wwox* knockouts at E16.5, in agreement with the reduced neurogenesis (Additional file 1: Figure S5c).

During murine neocortical development, neural progenitor cells (NPCs) undergo proliferation in the ventricular and subventricular zones (VZ and SVZ, respectively) between E11.5 and E16.5 to generate different projection neuron subtypes, and the proliferative activity of NPCs declines after E16.5 [24]. The newly born neurons henceforth exit the cell cycle and migrate to the outer zone of neocortex to develop into mature neurons. Following a pulse of bromodeoxyuridine (BrdU) to pregnant dams for labeling the actively proliferating cells in embryos at E16.5, our results showed that most BrdU^+^ neurons born shortly after E16.5 have migrated from the VZ and SVZ to the cortical plate (CP) of *Wwox*^+/+^ and *Wwox*^+/−^ neocortex at birth (Additional file 1: Figure S6a). In comparison with their littermates, increased BrdU^+^ neurons were found throughout the neocortex of *Wwox*^−/−^ newborn mice, with a large proportion of the nascent neurons still residing in the VZ and SVZ (Additional file 1: Figure S6a). More Ki-67^+^ proliferating neurons were also observed in the VZ and SVZ of *Wwox*^−/−^ neocortex at birth (Additional file 1: Figure S6b). These results suggest that *Wwox*^−/−^ neurons that still have high proliferative activity after E16.5 may be in a less differentiated state and have poor mobility during neocortical development.

**Fig. 6:**
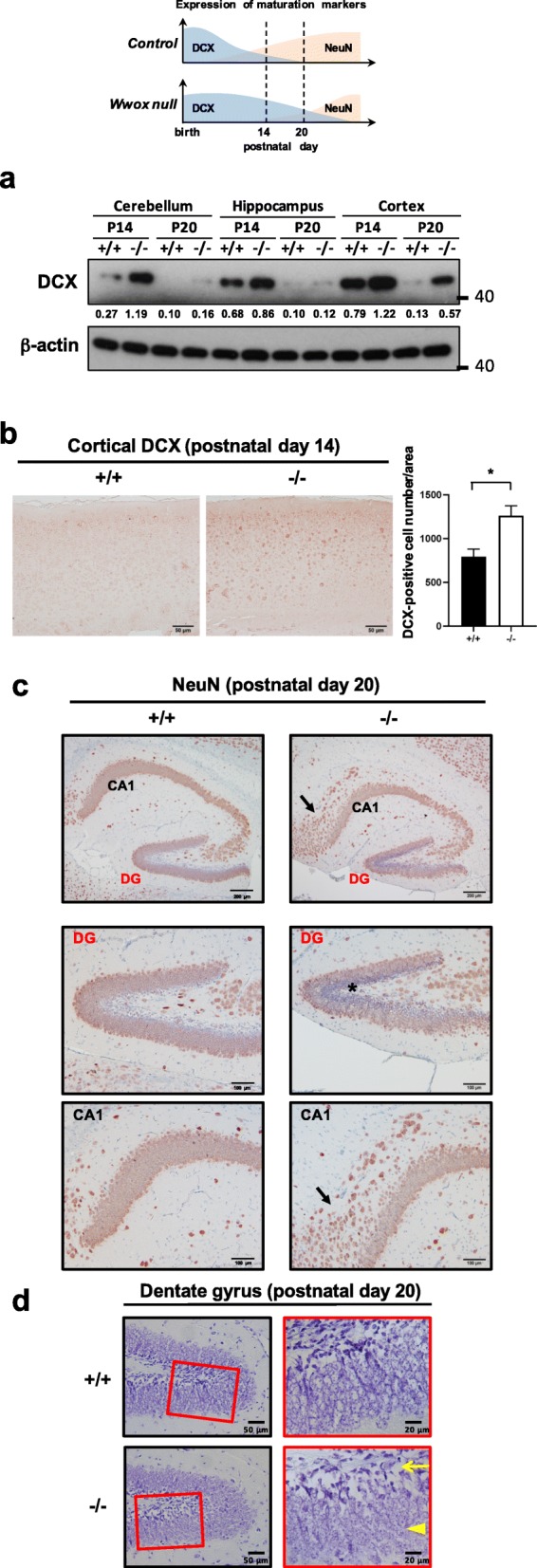
Defective CNS development in *Wwox* knockout mice. **a** Cerebellum, hippocampus and cerebral cortex protein samples from *Wwox*^*+/+*^ and *Wwox*^*−/−*^ mice at postnatal day 14 or 20 were examined for the expression levels of an early neuronal differentiation marker DCX by western blotting. β-actin was used as an internal control. Quantitative densitometry of the immunoblots was performed and the numbers depict the ratio of DCX to β-actin protein level in the brain tissues. **b** Immunohistochemistry was performed to determined DCX expression in the cerebral cortex of *Wwox*^*+/+*^ and *Wwox*^*−/−*^ mice at postnatal day 14 (*N* = 5). Scale bars = 50 μm. The numbers of DCX-positive cells in five representative subareas of *Wwox*^*+/+*^ and *Wwox*^*−/−*^ cerebral cortex tissue sections were quantified (right panel). The results are expressed as means ± SEM. *P < 0.05. **c** Sagittal brain sections of *Wwox*^*+/+*^ and *Wwox*^*−/−*^ mice at postnatal day 20 were immunostained with anti-NeuN. Compared with the age-matched control littermates, a large proportion of neurons in the hippocampal dentate gyrus (DG) of *Wwox*^*−/−*^ mice showed absent expression of a mature neuronal marker NeuN (black star). Dispersed distribution of NeuN-positive neurons in the hippocampal CA1 region of *Wwox*^*−/−*^ brain was observed (black arrow). The lower panel pictures of dentate gyrus and CA1 regions are the magnified images from the boxed area in the upper panel. The representative images of six independent experiments are shown. Scale bars = 200 (upper) or 100 μm (lower). DG, dentate gyrus. **d** Cresyl violet staining of sagittal brain sections revealed a decreased cell density in the subgranule zone (yellow arrow) and a less orderly arrangement of granule neurons (yellow arrowhead) in the hippocampal dentate gyrus of *Wwox*^*−/−*^ mice at postnatal day 20. The right panel pictures are the magnified images from the boxed area in the left panel. The representative images of five independent experiments are shown. Scale bars = 50 (left) or 20 μm (right)

To further verify whether the neuronal development in *Wwox*^−/−^ mice lags behind the wild-type littermates, the expression of DCX protein, an early differentiation marker expressed by NPCs and immature neurons, was examined in these mice. The DCX expression starts to decline as the precursor cells differentiate into mature neurons. Compared with the DCX protein levels in *Wwox*^+/+^ and *Wwox*^+/−^ mice, our results showed that DCX was still highly expressed in *Wwox*^−/−^ brain tissues at postnatal day 14 (Fig. 6a, b and Additional file 1: Figure S7). During neurogenesis, DCX protein downregulation in the developing neurons is followed by the expression of a mature neuronal marker NeuN. In comparison with the neurons in the dentate gyrus of wild-type hippocampus that strongly expressed NeuN, many cells in the dentate gyrus of *Wwox*^−/−^ mice showed absent expression of NeuN at postnatal day 20 (Fig. 6c). Moreover, the neuronal cells in the CA1 region of *Wwox*^−/−^ hippocampus were abnormally dispersed (Fig. 6c). Disorganization of neuronal cells in the dentate gyrus was also observed in the cresyl violet-stained brain section of *Wwox*^−/−^ mice (Fig. 6d). Neuronal heterotopia (ectopic neurons) could be found in *Wwox*^−/−^ mouse brain cortex (Additional file 1: Figure S8). Neuronal cell apoptosis was observed in the brain tissues of *Wwox*^−/−^ mice (Additional file 1: Figure S9). Together, in agreement with a recent study using a human neural progenitor cell culture system [34], our results indicate that loss of *Wwox* causes disorders in neuronal migration and development and brain malformations in mice.

### GSK-3β inhibition ameliorates the hypersusceptibility to epileptic seizure due to *Wwox* loss in mice

Abnormally migrated neurons form reorganized neuronal networks that create hyperexcitable tissue in the brain and exhibit altered cellular physiologies. Neuronal migration abnormalities during development and heterotopia have been suggested to be associated with increased neuronal excitability, epilepsy and mild to moderate mental retardation in humans and mice [25, 49, 54]. Similar to the spontaneous *Wwox* mutation in *lde*/*lde* rats, a patient with a homozygous *WWOX* nonsense mutation has been reported to display a phenotype of growth retardation, microcephaly, epilepsy, retinal degeneration and early death [2, 57]. In our generated *Wwox*^−/−^ mice, spontaneous epileptic seizures were commonly observed after postnatal day 12. Seizures were frequently induced by mild stressors including noise, strobe lights and novel cage during routine handling (Additional file 2: Movie S1).


**Additional file 2**: Video of spontaneous epileptic seizure.


**Fig. 7:**
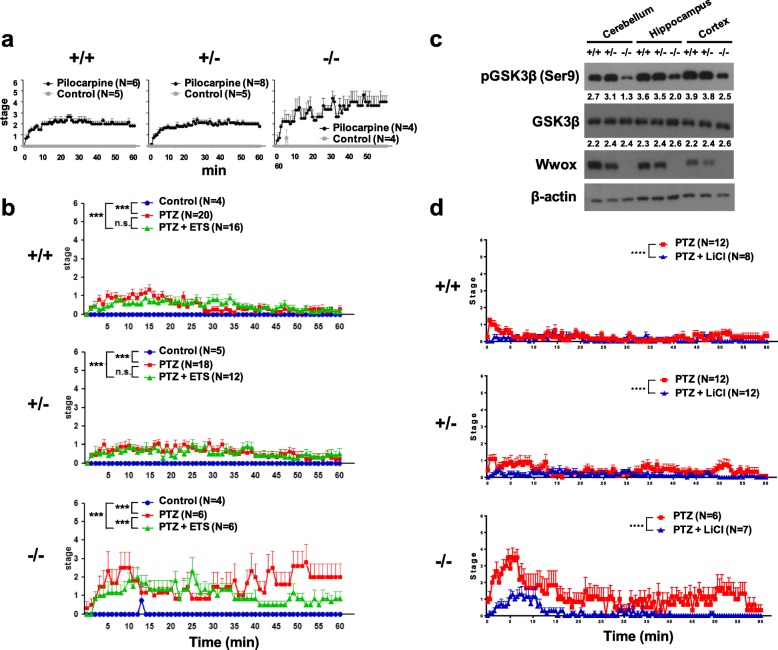
Increased GSK3β activity in the brain tissues leads to hypersusceptibility to drug-induced seizure in *Wwox* knockout mice. **a** *Wwox*^*−/−*^ mice showed increased susceptibility to seizure induction by the injection of pilocarpine (50 mg/kg), a muscarinic receptor agonist. Behavioral scoring of seizure severity according to the Racine’s scale [55] in three genotypes of mice for 60 min is presented. **b** Higher seizure activity was observed in *Wwox*^*−/−*^ mice after injection of PTZ (30 mg/kg), a GABAergic receptor antagonist, as compared with *Wwox*^*+/+*^ and *Wwox*^*+/−*^ mice. Pretreatment of ethosuximide (ETS, 150 mg/kg) suppressed PTZ-induced seizure activity in *Wwox*^*−/−*^ mice. **c** Increased activation of GSK3β was determined in the cerebellum, hippocampus and cerebral cortex of *Wwox*^*−/−*^ mice at postnatal day 20, as evidenced by dephosphorylation of GSK3β at Ser9. β-actin was used as an internal control in western blotting. Quantitative densitometry of the immunoblots was performed, and the numbers depict the ratio of phosphorylated or total GSK3β to β-actin protein level in the brain tissues. The representative results of four independent experiments are shown. **d** Pretreatment of a GSK3β inhibitor LiCl (60 mg/kg) suppressed PTZ-induced seizure activity in *Wwox*^*−/−*^ mice. The results are expressed as means ± SEM. n.s., non-significant. ***P < 0.001

To further investigate the enhanced epileptogenesis in *Wwox*^−/−^ mice, we tested convulsant agent-induced seizure models using a muscarinic receptor agonist pilocarpine and a GABAergic receptor antogonist PTZ. After intraperitoneal injection of pilocarpine (50 mg kg^− 1^) or PTZ (30 mg kg^− 1^), seizure severity was monitored according to the Racine’s scale [55]. Compared with the *Wwox*^*+/+*^ and *Wwox*^*+/−*^ littermates, we found that *Wwox*^*−/−*^ mice showed enhanced susceptibilities to the stimulation of either pilocarpine (Fig. 7a) or PTZ (Fig. 7b), and developed to a series of generalized tonic-clonic seizures immediately after injection. Half of the pilocarpine- or PTZ-injected *Wwox*^*−/−*^ mice evolved into status epilepticus (SE, defined as three or more tonic-clonic seizures during 1-h observation). SE was not observed in *Wwox*^*+/+*^ and *Wwox*^*+/−*^ mice. Pretreatment of an antiepileptic drug ethosuximide suppressed PTZ-induced seizure in *Wwox*^*−/−*^ mice (Fig. 7b), although ethosuximide pretreatment had no effects on the behavior changes in *Wwox*^*+/+*^ and *Wwox*^*+/−*^ mice treated with a low dose of PTZ.

WWOX has been shown to interact with and inhibit GSK3β, thereby increasing the microtubule assembly activity of Tau and promoting neurite outgrowth in human neuroblastoma SH-SY5Y cells [65]. To investigate whether the enhanced epileptogenesis in *Wwox*^−/−^ mice is due to increased GSK3β activation in neuronal cells, we determined dephosphorylation of GSK3β at Ser9 (active GSK3β) in *Wwox*^−/−^ mouse cerebellum, hippocampus and brain cortex by western blotting (Fig. 7c). Injection of a potent GSK3β inhibitor lithium chloride significantly suppressed PTZ-induced epileptic seizure in *Wwox*^*−/−*^ mice (Fig. 7d). Together, these results suggest an important role of GSK3β in the hypersusceptibility to epileptic seizure induction due to *Wwox* loss in neuronal cells.

## Discussion

In spite of its putative function as a tumor suppressor, *Wwox* expresses abundantly in mouse developing nervous system [19]. In this study, we applied a mouse genetics approach and demonstrated that *Wwox* deficiency in mice leads to neurodevelopmental deficits and neurodegeneration that resemble human neuropathologic features. First, severe hypomyelination with atrophy of optic tract and white matter fiber tracts in *Wwox*^−/−^ mouse brain recapitulates the clinical findings in patients with homozygous mutations in *WWOX* gene. Of note, our electron microscope images revealed Schwann cell apoptosis and axon demyelination and degeneration in *Wwox*^*−/−*^ sciatic nerves. Since normal conductance of nerve impulses depends on the insulating properties of myelin sheath surrounding the nerve fiber, severe hypomyelination in the central and peripheral nervous systems may cause the behavioral deficits including poor balance, motor incoordination and gait ataxia in *Wwox*^*−/−*^ mice. Myelin is composed of lipid-rich substance generated by oligodendrocytes in central nervous system (CNS) and by Schwann cells in PNS. The major protein content of CNS myelin includes myelin basic protein (MBP), myelin oligodendrocyte glycoprotein (MOG), myelin-associated glycoprotein (MAG) and proteolipid protein (PLP). MOG is unique to the CNS myelin. In addition to MBP and MAG, the PNS myelin contains abundant myelin protein zero (MPZ) that is absent in the CNS and is involved in holding together the multiple concentric layers of PNS myelin sheath. A recent study has reported a significantly decreased number of mature oligodendrocytes and reduced MBP expression in the cerebral cortex of *lde* rats with spontaneous *Wwox* mutation [61]. Mutations in the myelin proteins, such as PLP and MPZ, are associated with the neuropathic disorders in patients with Pelizaeus-Merzbacher disease and Charcot-Marie-Tooth disease, respectively [40]. Inflammatory responses against MBP, MOG and MAG are known to cause demyelinating diseases. Whether *Wwox* deficiency leads to myelin protein deficits or neurodegenerative autoimmune diseases is unknown. Moreover, WWOX has been suggested to be associated with lipid metabolism [4, 31, 36, 39]. Whether WWOX regulates myelin formation via controlling lipid biosynthesis and metabolism and supports cell survival in oligodendrocytes and Schwann cells needs further investigation.

Second, we show here that *Wwox* deficiency in mice leads to marked foliation defects and loss of Purkinje cells along with granular cell apoptosis in the cerebellum (Fig. 5). Cerebellar hypoplasia and aberrant foliations in vermian lobules VI and VII have been shown to be associated with defective Wnt/β-catenin signaling in a mouse model of loss of function of *Ahi1*, a Joubert syndrome-associated gene [38]. Joubert syndrome is an autosomal recessive neurodevelopmental disorder characterized by agenesis of cerebellar vermis, neonatal hypotonia, ataxia, developmental delay, and cognitive disabilities including autism and mental retardation. Smad2 depletion in mice also caused cerebellar foliation anomalies and ataxia [66]. WWOX has been suggested to regulate β-catenin and Smad-driven promoter activities in Wnt and TGF-β signaling, respectively [5, 28, 29]. Because the survival of cerebellar granular cells largely depends on their synaptic connection with Purkinje cells [45], whether WWOX prevents Purkinje cell degeneration, thereby supporting granular cell growth during cerebellar development via regulating Wnt/β-catenin and TGF-β/Smad2 signaling pathways remains to be determined.

Cerebellar ontogenesis is regulated by lipophilic hormones, including thyroid hormone and sex steroids [6, 21, 33]. In perinatal hypothyroidism, the growth and branching of Purkinje cell dendrites are greatly reduced. Thyroid hormone deficiency also causes delayed migration of granular cells to the internal granular cell layer and defective synaptic connection within the cerebellar cortex [33]. WWOX has been shown to be highly expressed in the secretory epithelial cells of hormonally regulated organs including breast, ovary, testis and prostate, and targeted deletion of *Wwox* in mouse mammary gland leads to impaired mammary ductal development [1, 53]. WWOX expression is relatively strong in human, rat and mouse neural tissues, and varies according to the location [19, 34, 53, 61]. Notably, WWOX may interact with steroid hormone 17β-estradiol via its NSYK (Asn-Ser-Tyr-Lys) motif in the *C*-terminal short-chain alcohol dehydrogenase/reductase domain for neuroprotection [42]. Whether WWOX acts as a receptor for steroid hormones for initiating neuroprotective signaling pathways and promoting cerebellum development is unclear. The functional role of *Wwox* in a particular cell type needs to be further analyzed using conditional tissue-specific knockout mouse models.

Third, we identify a crucial role of WWOX in neurogenesis and neocortical development. Mammalian CNS development is achieved by proliferation of NPCs followed by their transition from a proliferative state to differentiation. In the developing cerebral cortex, NPCs exit the cell cycle in the VZ and SVZ, whereafter the postmitotic neurons migrate towards the outer zone of neocortex to form laminated cortical layers. At birth, a large number of the postmitotic neurons born around E16.5 have migrated to the CP and developed into mature neurons in *Wwox*^+/+^ and *Wwox*^+/−^ mouse neocortex, whereas the *Wwox*^*−/−*^ neocortical neurons display aberrant progenitor proliferation and migration and are less differentiated. Our findings raise some new questions. For instance, it is unclear whether the deficits in neuronal migration and differentiation are associated with the aberrant proliferation of *Wwox*^*−/−*^ neocortical progenitor neurons. Also, whether WWOX regulates the switch from progenitor proliferation to migration in the developing brain is unknown.

The development of mammalian cerebral cortex and hippocampus involves neuronal proliferation, migration, and synaptic refinement within the neuronal circuitry. Neuronal migration deficits during development may lead to malformations of cerebral neocortex and hippocampus that greatly increase neuronal excitability and the risk of seizures [49, 52]. *Wwox*^*−/−*^ mice exhibit cerebral malformations consisting of middle interhemispheric fusion, cortical heterotopia and neuronal disorganization in the hippocampal CA1 region and display increased susceptibilities to convulsant-induced seizures. The aberrant positioning of neurons in *Wwox*^*−/−*^ neocortex and hippocampus may cause reorganization of neuronal networks and alteration of cellular physiology that create hyperexcitable tissue. Foci of aberrantly migrated neurons and cortical dysplasias have been known to be associated with pharmacologically intractable epilepsies. Administration of GSK3β inhibitor lithium chloride effectively ameliorated the seizure susceptibility in *Wwox*^*−/−*^ mice, and its efficacy is better than the commonly used anticonvulsant drug ethosuximide. Lithium is a widely used mood stabilizer in the treatment of bipolar and depressive disorders. Administration of lithium in mice has been demonstrated to attenuate PTZ-induced clonic seizure [10], and rescue Wnt-dependent cerebellar midline fusion and neurogenesis deficits early in development [38]. Lithium treatment has also been shown to induce β-catenin-mediated myelin gene expression in mouse Schwann cells and enhance remyelination of the injured peripheral nerves in mice [47]. GSK3β signaling plays key roles in the regulation of neurogenesis, neuronal polarization and axon growth during neural development [30]. WWOX interacts with GSK3β and suppresses GSK3β-mediated Tau phosphorylation for promoting retinoic acid-induced microtubule assembly activity of Tau and neurite outgrowth in SH-SY5Y cells [65]. WWOX also binds to Tau via its *C*-terminal short-chain alcohol dehydrogenase/reductase domain for preventing Tau hyperphosphorylation and neurofibrillary tangle formation [59]. Without WWOX, protein aggregation cascade starting from TRAPPC6AΔ, TIAF1 and SH3GLB2 may cause APP degradation and aggregation of amyloid β and Tau in neurons [15, 20]. Downregulation of WWOX protein expression has been observed in the hippocampal neurons in patients with Alzheimer’s disease [59]. Future experiments can now be directed at determining the regulation of GSK3β activity by WWOX in neural development and neurodegeneration. Whether lithium treatment can rescue the deficits in neuronal migration and differentiation during development in *Wwox*^*−/−*^ mice remains to be studied.

In summary, *Wwox* gene ablation causes severe neurodevelopmental and neurodegenerative disorders and convulsive seizures in mice. Most importantly, *Wwox* knockout mouse models recapitulate the key pathological features of human neuropathies and can be considered a valuable research tool for delineation of molecular pathogenesis and development of therapeutic strategies for refractory epilepsy. Future studies, as well as more evaluations, will be needed to test whether GSK3β inhibitors may be promising candidates for treatment of human neurological disorders due to loss or dysfunction of WWOX.

## Additional file

**Additional file 1:** Supplementary Methods. **Figure S1.** *Wwox* gene deletion causes microcephaly and abnormal brain morphology in mice. **Figure S2.** Immunofluorescence staining of sagittal cerebellar tissue sections from *Wwox*^+/+^ and *Wwox*^-/-^ mice using an anti-calbindin monoclonal antibody. **Figure S3.** Increased apoptotic cells in the granular layer of *Wwox*^-/-^ mouse cerebellum. **Figure S4.** A lack of full separation of cerebral hemispheres is evident in *Wwox*^-/-^ mice. **Figure S5.** The development of *Wwox*^-/-^ mouse central nervous system is defective during the embryonic stages. **Figure S6.** *Wwox*^-/-^ mouse neocortical neurons retain high proliferative activity after E16.5 and have poor mobility during development. **Figure S7.** *Wwox* loss leads to the increased DCX protein levels in mouse brain tissues at postnatal day 14. **Figure S8.** Neuronal heterotopia can be observed in the cortex of *Wwox* knockout mouse brain. **Figure S9.** Increased neuronal apoptosis is detected in *Wwox* knockout mouse brain.

## Data Availability

All data generated and/or analyzed in this study are included in this published article and its supplementary information files.

## Abbreviations

AEC: 3-amino-9-ethylcarbazole
ANOVA: Analysis of variance
Ax: Axons
BrdU: Bromodeoxyuridine
CNS: Central nervous system
CP: Cortical plate
DCX: Doublecortin
DG: Dentate gyrus
En: Endoneurium
ETS: Ethosuximide
GSK3β: Glycogen synthase kinase 3β
GTCS: Generalized tonic-clonic seizure
H&E: Hematoxylin and eosin
i.p.: Intraperitoneally
LFB: Luxol fast blue
LiCl: Lithium chloride
MAG: Myelin-associated glycoprotein
MBP: Myelin basic protein
MEF: Mouse embryonic fibroblasts
MOG: Myelin oligodendrocyte glycoprotein
MPP^+^: 1-methyl-4-phenyl-pyridinium
MPZ: Myelin protein zero
My: Myelin sheath
NPCs: Neural progenitor cells
NSYK: Asn-Ser-Tyr-Lys
PLP: Proteolipid protein
PNS: Peripheral nervous system
PTZ: Pentylenetetrazol
Sc: Schwann cell
SE: Status epilepticus
SEM: Standard error of the mean
SVZ: Subventricular zones
Tc-MEPs: Transcranial motor evoked potentials
TUNEL: Terminal deoxynucleotidyl transferase dUTP nick end labeling
VZ: Ventricular zones
WD1: *Wwox* gene exon-1 deletion
WD234: *Wwox* gene exon-2/3/4 deletion
WWOX: WW domain-containing oxidoreductase
