# Supplementary material for: Wwox deficiency leads to neurodevelopmental and degenerative neuropathies and glycogen synthase kinase 3β-mediated epileptic seizure activity in mice
Source: Acta Neuropathol Commun. 2020 Jan 30;8:6. doi: 10.1186/s40478-020-0883-3 (PMC6990504; doi:10.1186/s40478-020-0883-3)
Supplement: Supplementary file 2 — Additional file 1: Supplementary Methods. Figure S1. Wwox gene deletion causes microcephaly and abnormal brain morphology in mice. Figure S2. Immunofluorescence staining of sagittal cerebellar tissue sections from Wwox+/+ and Wwox-/- mice using an anti-calbindin monoclonal antibody. Figure S3. Increased apoptotic cells in the granular layer of Wwox-/- mouse cerebellum. Figure S4. A lack of full separation of cerebral hemispheres is evident in Wwox-/- mice. Figure S5. The development of Wwox-/- mouse central nervous system is defective during the embryonic stages. Figure S6. Wwox-/- mouse neocortical neurons retain high proliferative activity after E16.5 and have poor mobility during development. Figure S7. Wwox loss leads to the increased DCX protein levels in mouse brain tissues at postnatal day 14. Figure S8. Neuronal heterotopia can be observed in the cortex of Wwox knockout mouse brain. Figure S9. Increased neuronal apoptosis is detected in Wwox knockout mouse brain. [file 40478_2020_883_MOESM1_ESM.pdf]

Wwox deficiency leads to neurodevelopmental and degenerative neuropathies and glycogen synthase kinase 3β-mediated epileptic seizure activity in mice

Ya-Yun Cheng[1,†], Ying-Tsen Chou[2], Feng-Jie Lai[3,4,†], Ming-Shiou Jan[5], Tsung-Hao Chang[2], I-Ming Jou[6], Pei-Shiuan Chen[1], Jui-Yen Lo[2], Shiang-Suo Huang[7], Nan-Shan Chang[8,9,10], Yung-Tsai Liou[11], Po-Chih Hsu[1], Hui-Ching Cheng[2], Yee-Shin Lin[9,11], Li-Jin Hsu[1,2,9,12,*]

[1]Department of Medical Laboratory Science and Biotechnology, College of Medicine, National Cheng Kung University, Tainan, Taiwan

[2]Institute of Basic Medical Sciences, College of Medicine, National Cheng Kung University, Tainan, Taiwan

[3]Department of Dermatology, Chi Mei Medical Center, Tainan, Taiwan

[4]Center for General Education, Southern Taiwan University of Science and Technology, Tainan, Taiwan

[5]Institute of Microbiology and Immunology, Chung Shan Medical University, Taichung, Taiwan

[6]Department of Orthopaedics, College of Medicine, National Cheng Kung University, Tainan, Taiwan

[7]Department of Pharmacology, Chung Shan Medical University, Taichung, Taiwan

[8]Institute of Molecular Medicine, College of Medicine, National Cheng Kung University, Tainan, Taiwan

[9]Center of Infectious Disease and Signaling Research, College of Medicine, National Cheng Kung University, Tainan, Taiwan

[10]Department of Neuroscience and Physiology, SUNY Upstate Medical University, Syracuse, New York, USA

[11]Department of Microbiology and Immunology, College of Medicine, National Cheng Kung University, Tainan, Taiwan

[12]Research Center for Medical Laboratory Biotechnology, College of Medicine, National Cheng Kung University, Tainan, Taiwan

[†]These authors contributed equally to this work.

[*]Corresponding author.

Supplementary Methods

Generation of *Wwox* gene knockout mice, Southern blotting and PCR genotyping.

Strategies for making *Wwox* knockout mice are depicted in the diagrams below. Blue rectangles represent *Wwox* exons. Neo indicates the neomycin selection cassette flanked by *FRT* recombination sites (purple circle). The BAC clone containing genomic DNA coding for mouse *Wwox* was obtained from Geneservice (Cambridge, UK) and used for construction of the targeting vectors using a "recombineering" method. The "recombineering" method can generate large targeting vectors with very long homologous arms derived from the BAC clone, which would greatly facilitate the targeting efficiency in embryonic stem (ES) cells (Copeland et al., 2001). We designed insertion of *LoxP* sites (green triangles) to *Wwox* gene for targeting either exon 1 (WD1) or exon 2/3/4 (WD234), and performed *Cre*/*LoxP*-mediated excision of the targeted sequences in 129/Sv mouse ES cells. The targeted ES cells were selected and injected into C57BL/6 blastocysts to generate chimeric animals, and germ-line transmission of the knockout allele was obtained by breeding the male chimera with female C57BL/6 mice (Yu et al., 2004). Increased C57BL/6 genetic background of the mice carrying *Wwox* null allele was obtained by successive backcrosses of the $Wwox^{+/-}$ progenies to wild-type C57BL/6 mice. Heterozygous mice were then interbred to obtain $Wwox^{+/+}$, $Wwox^{+/-}$, and $Wwox^{-/-}$ progenies.

Mouse embryonic fibroblasts (MEFs) were derived from ~E16.5 fetuses. Genomic DNA samples isolated from MEFs were digested with ScaI. Agarose gel electrophoresis and Southern blotting were carried out to confirm the knockout mouse genotypes. The sequences of primer sets used for generating probes for Southern blot analysis were as follows: forward 5'-TTACTAACATTTTTGCAAAG and reverse 5'-CCATCTTGGAACCACTGC for WD1; and forward 5'-GGGCATGGGACGAAACTG and reverse 5'-GCAGGAGCTTGGCTTTGA for WD234. The probes were labelled with digoxigenin and used for hybridization using a standard protocol described previously (Yu et al., 2004). The hybridization signals on the positively charged nylon membrane for each probe were detected using an enzyme-conjugated anti-digoxigenin antibody and exposed to X-ray films after the addition of the chemiluminescent substrate to the blots.

For PCR genotyping, tail genomic DNA was isolated from mice at postnatal day 5~10. The primer sequences used for PCR were:

Forward (WD1-F) 5'-TGAGCTTGGGAGAAGTGGGTACTTTG,

Reverse 1 (WD1-R1) 5'-AGCTCTATACTATACTGGCTGGCTGG,

Reverse 2 (WD1-R2) 5'-AGGTGTTGGAGACTTCTCCACTGCTA for WD1; and

Forward (WD234-F) 5'-GCTCTGTGAGACCATTTGGACAGTGT,

Reverse 1 (WD234-R1) 5'-CTTGATTCTGCTGCCTCTGCTTCCTA,

Reverse 2 (WD234-R2) 5'-CGAGAGAAGGAAGCCTGTTATCTAGA for WD234.

The PCR condition was 1 cycle of 95°C, 5 min, 40 cycles of 95°C, 30 sec, 60°C, 30 sec, and 72°C, 1 min, and 1 cycle of 72°C, 10 min. PCR genotyping using the specific primer set for WD1 generated a 593-bp product from the wild-type (WT) allele and a 450-bp product from the *Wwox* null allele (Figure S1a). For WD234, PCR products of 696-bp and 505-bp were detected for the WT and *Wwox*-deleted alleles, respectively (Figure S2b). Neonatal mice at postnatal day 5~12 from $Wwox^{+/-}$ intercrosses were collected for genotyping and the percentages of $Wwox^{-/-}$ mice were 5.1% and 6.5% for WD1 (N=177) and WD234 (N=785), respectively.

Measurement of brain water content

Mouse brain water content was measured as described in a previous study (Lin et al., 2000) with slight modifications. Briefly, mice were sacrificed in a CO_2 euthanasia chamber, and the brains were immediately removed and measured as wet weight (WW). The brains were cut into 2-mm sections and dried at 85°C for 48 hours to determine the dry weight (DW). Brain water content was calculated as the following:

Brain water content (%) = [(WW - DW)/WW] x 100%

References

Copeland NG, Jenkins NA, Court DL. Recombineering: a powerful new tool for mouse functional genomics. Nat Rev Genet 2001; 2(10):769-79.

Yu IS, Lin SR, Huang CC, Tseng HY, Huang PH, Shi GY, Wu HL, Tang CL, Chu PH, Wang LH, Wu KK, Lin SW. TXAS-deleted mice exhibit normal thrombopoiesis, defective hemostasis, and resistance to arachidonate-induced death. Blood 2004;104(1):135-42.

Lin W, Venkatesan R, Gurleyik K, He YY, Powers WJ, Hsu CY. An absolute measurement

of brain water content using magnetic resonance imaging in two focal cerebral ischemic rat models. J Cereb Blood Flow Metab. 2000;20(1):37-44.

Diagrams for generating *Wwox* gene knockout mice

A. Targeting construct design

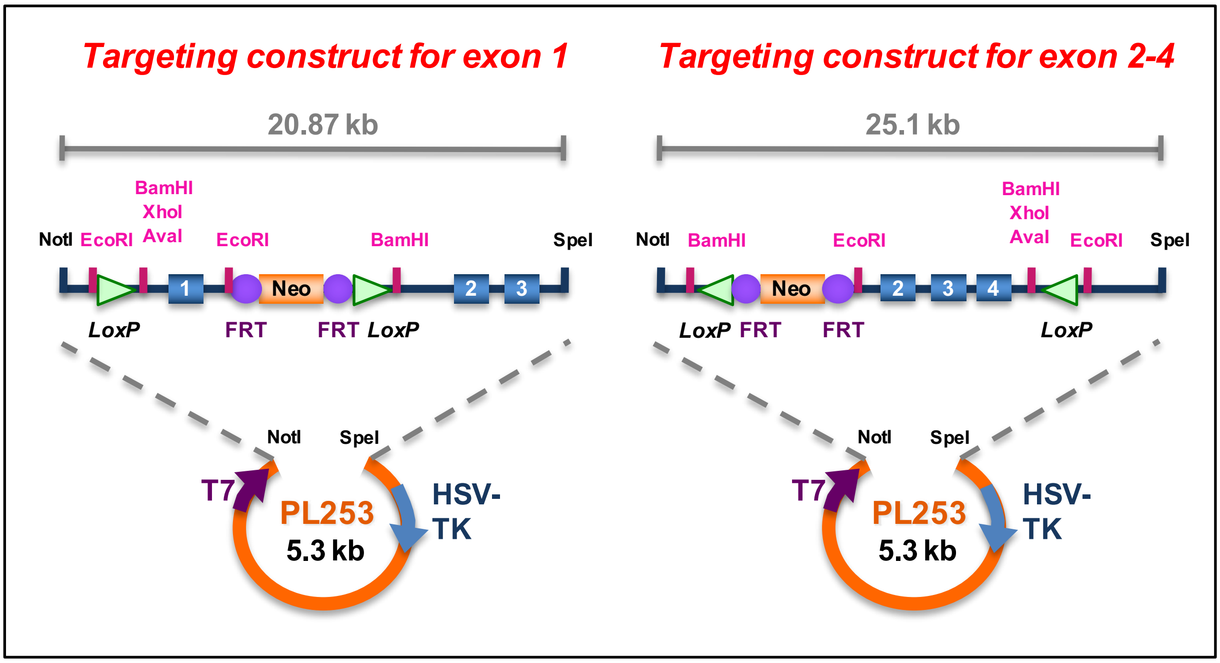

B. Targeting Wwox gene in mouse ES cells

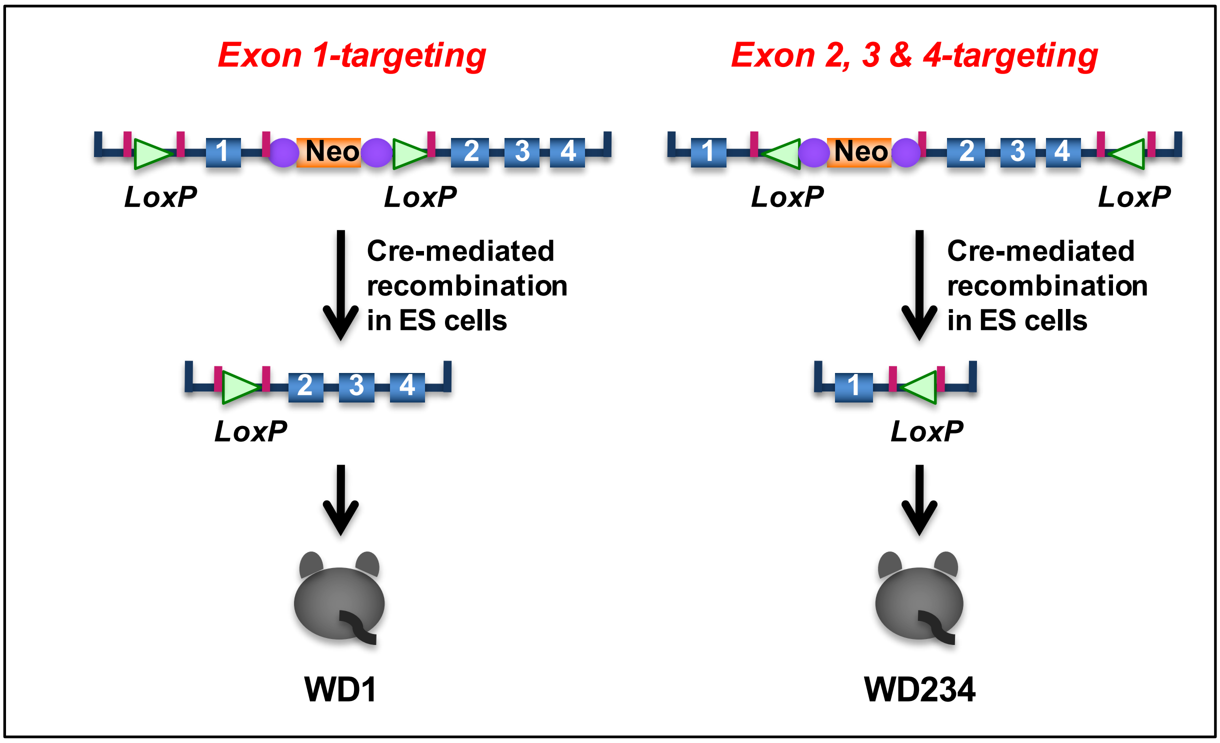

Supplementary Figure 1

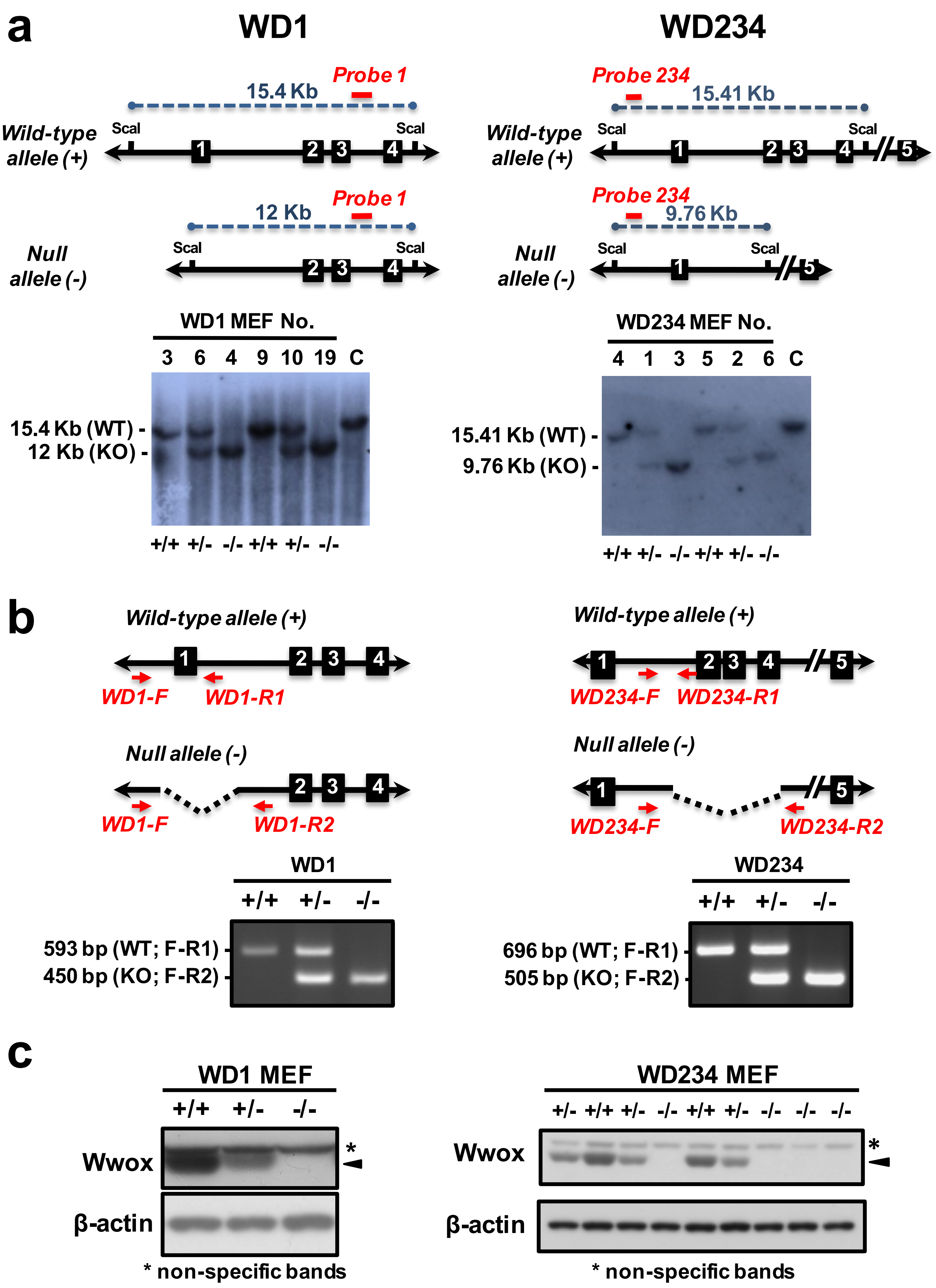

Supplementary Figure 1

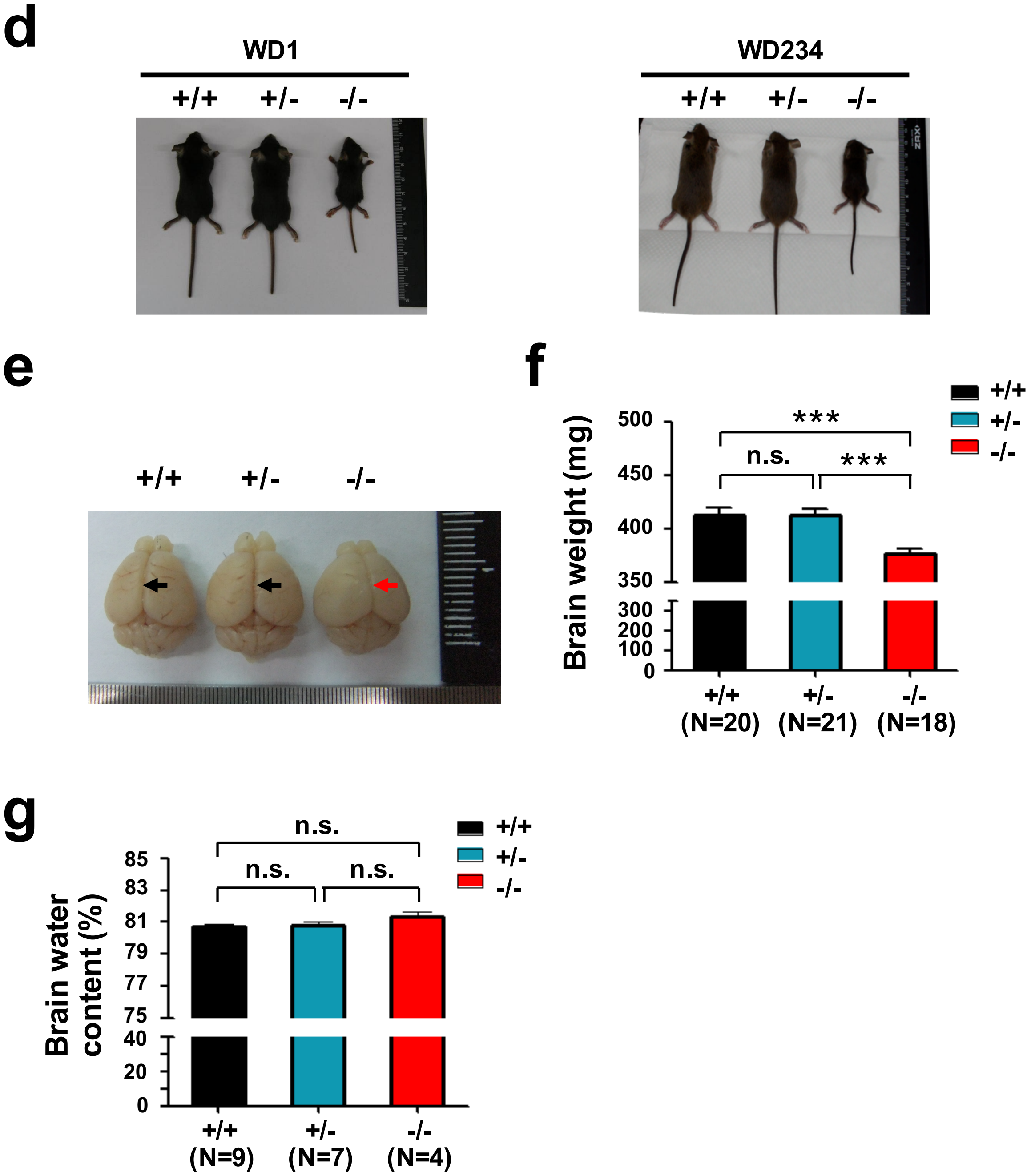

Supplementary Figure 1. *Wwox* gene deletion causes microcephaly and abnormal brain morphology in mice. **(a)** Southern blot analysis of ScaI-digested genomic DNA confirmed the deletion of *Wwox* exon 1 (WD1, left panel) or exon 2/3/4 (WD234, right panel) in MEFs. The primer sequences used for generating the digoxigenin-labeled probes (red) were 5'-ttactaacatttttgcaaag (forward) and 5'-ccatcttggaaccactgc (reverse) for WD1 (*probe 1*), and 5'-gggcatgggacgaaactg (forward) and 5'-gcaggagcttggctttga (reverse) for WD234 (*probe 234*). Black rectangles represent mouse *Wwox* exons. C, control DNA; KO, knockout; WT, wild-type. **(b)** Mouse tail DNA samples were subjected to PCR analysis for genotyping. The primer sequences used were as follows: WD1-F, 5'-tgagcttgggagaagtgggtactttg; WD1-R1, 5'-agctctatactatactggctggctgg; WD1-R2, 5'-aggtgttggagacttctccactgcta; WD234-F, 5'-gctctgtgagaccatttggacagtgt; WD234-R1, 5'- cttgattctgctgcctctgcttccta; WD234-R2, 5'-cgagagaaggaagcctgttatctaga. **(c)** Undetectable Wwox protein expression (46 kDa; arrowhead) was determined in the homozygous knockout ($Wwox^{-/-}$) WD1 and WD234 MEFs by western blotting. The asterisks indicate a non-specific band arising from the MEF lysates. β-actin was used as an internal control. **(d)** The body size of WD1 and WD234 mice at postnatal day 20. **(e)** Gross examination of $Wwox^{+/+}$, $Wwox^{+/-}$ and $Wwox^{-/-}$ mouse brains (WD1) at postnatal day 20. The arrows indicate the interhemispheric fissure. A partial middle interhemispheric fusion was observed in $Wwox^{-/-}$ mouse cerebrum (red arrow). **(f)** As compared with the $Wwox^{+/-}$ and $Wwox^{-/-}$ littermates, a significant reduction in $Wwox^{-/-}$ mouse brain weight was determined at postnatal day 20. **(g)** Comparable brain water contents of $Wwox^{+/+}$, $Wwox^{+/-}$ and $Wwox^{-/-}$ mouse brains were analyzed at postnatal day 20. Data are shown as means ± standard deviation. Comparisons were made by one-way analysis of variance (ANOVA). ***, $p < 0.005$; n.s., not significant.

Supplementary Figure 2

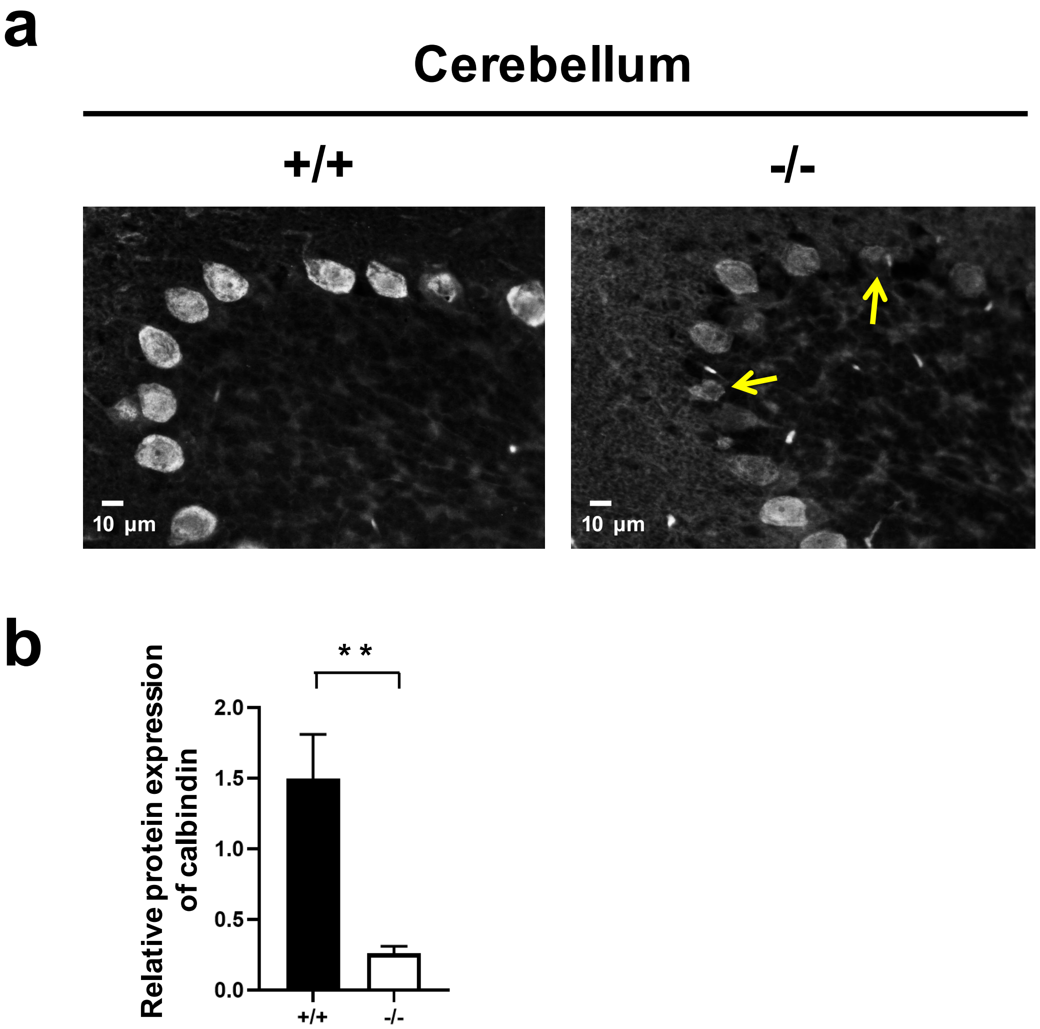

Supplementary Figure 2. Immunofluorescence staining of sagittal cerebellar tissue sections from *Wwox*$^{+/+}$ and *Wwox*$^{-/-}$ mice using an anti-calbindin monoclonal antibody (Sigma), followed by Alexa Fluor® 594-conjugated anti-mouse IgG antibody. **(a)** Compared with the wild-type littermates, diminished expression of calbindin was examined in the cerebellar Purkinje cells (yellow arrows) of *Wwox*$^{-/-}$ mice at postnatal day 20. Scale bars = 10 μm. **(b)** The quantitative intensity of calbindin-positive signal in *Wwox*$^{+/+}$ and *Wwox*$^{-/-}$ mouse cerebellar tissue sections was analyzed (N=4). The results are expressed as means ± SEM. $^{**}P < 0.01$.

Supplementary Figure 3

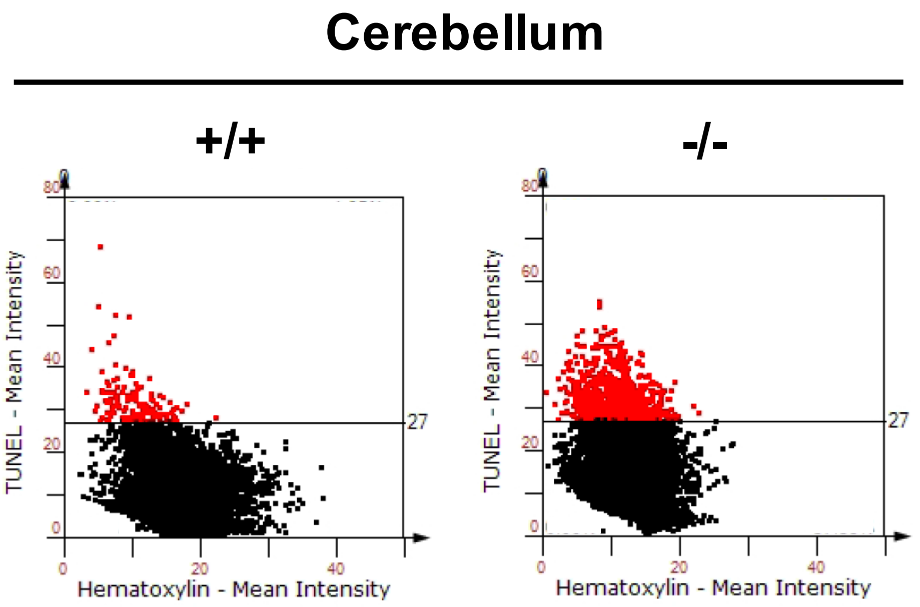

Supplementary Figure 3. Increased apoptotic cells in the granular layer of *Wwox*$^{-/-}$ mouse cerebellum. Sagittal cerebellar tissue sections from *Wwox*$^{+/+}$ and *Wwox*$^{-/-}$ mice at postnatal day 20 were used to perform terminal deoxynucleotidyl transferase dUTP nick end labeling (TUNEL) using a colorimetric ApopTag® Peroxidase In Situ Apoptosis Detection Kit (Millipore). After counterstaining with hematoxylin, the tissue sections were scanned and the increased intensity of TUNEL signal (Y-axis) in *Wwox*$^{-/-}$ mouse cerebellum was analyzed using a TissueFAXS Imaging System.

Supplementary Figure 4

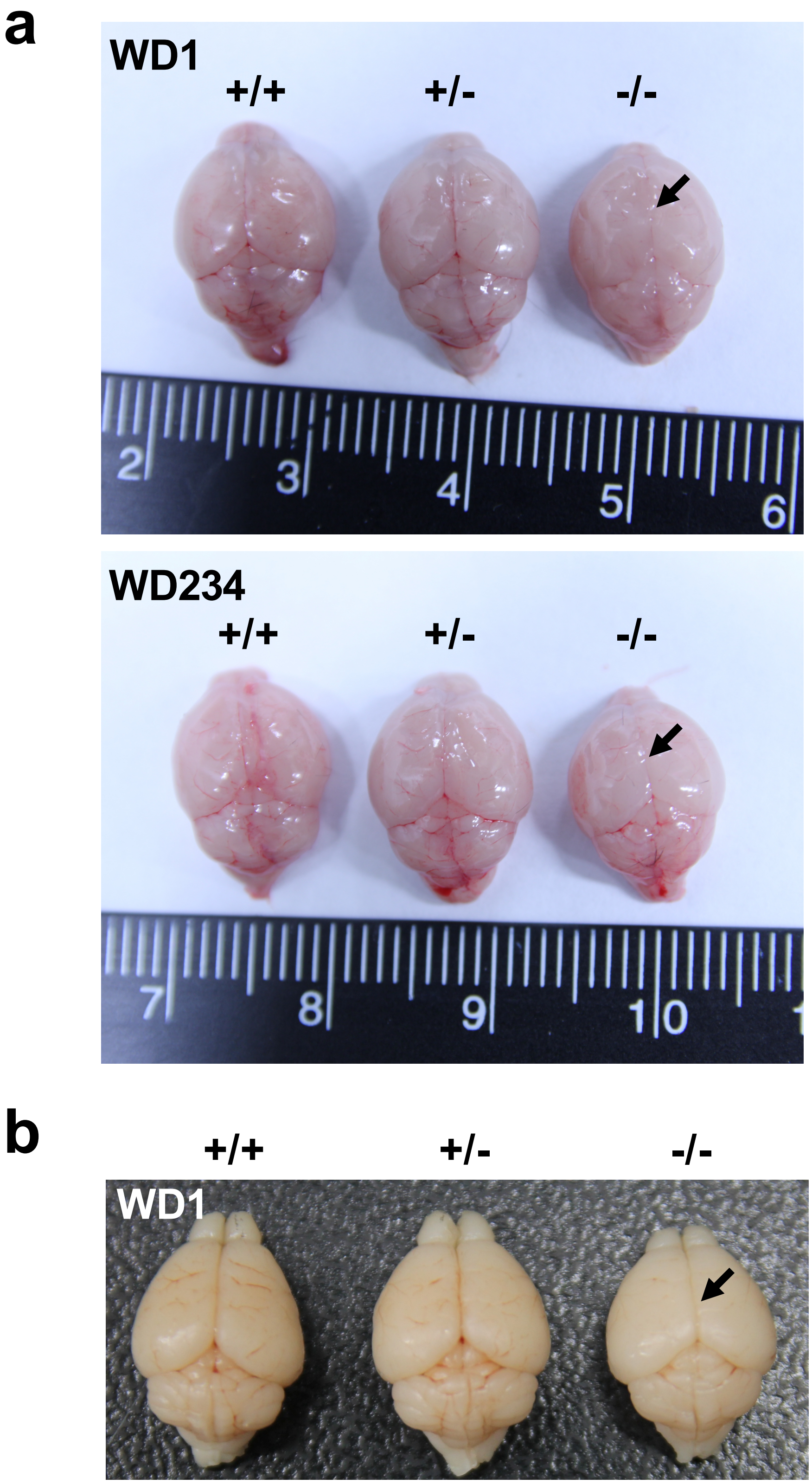

Supplementary Figure 4. A lack of full separation of cerebral hemispheres is evident in *Wwox*$^{-/-}$ mice. **(a)** Compared with *Wwox*$^{+/+}$ and *Wwox*$^{+/-}$ littermates, a partially formed interhemispheric fissure (arrow) was observed in both WD1 and WD234 *Wwox*$^{-/-}$ mice at postnatal day 20. **(b)** Similarly, a defective interhemispheric separation was observed in 3.7% formalin-fixed WD1 *Wwox*$^{-/-}$ mouse cerebrum at postnatal day 20 (data not shown for WD234).

Supplementary Figure 5

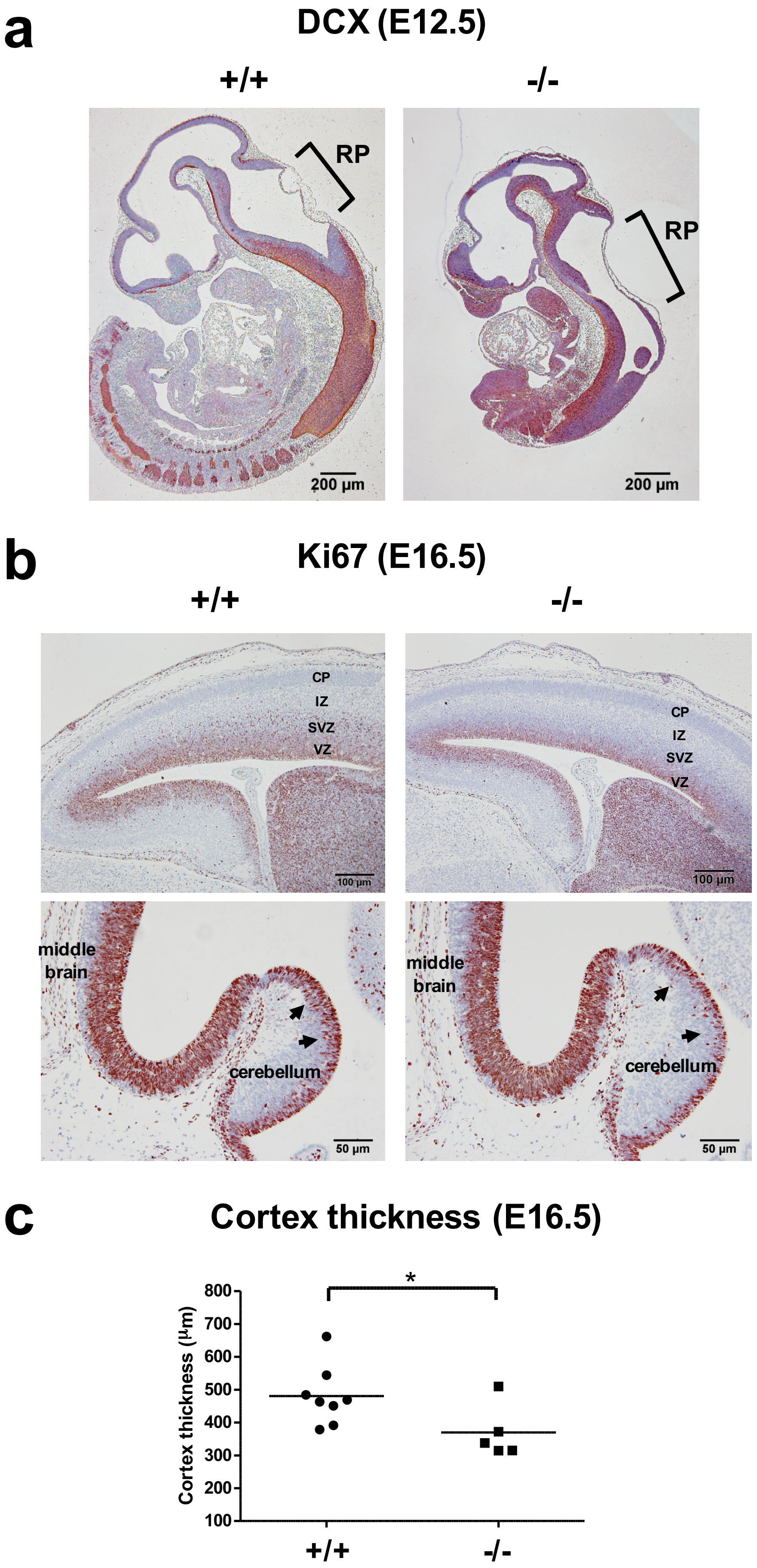

Supplementary Figure 5. The development of *Wwox*$^{-/-}$ mouse central nervous system is defective during the embryonic stages. **(a)** Sagittal sections of *Wwox*$^{+/+}$ and *Wwox*$^{-/-}$ mouse embryos (WD1) at E12.5 were immunostained with anti-doublecortin (DCX; an early neuronal differentiation marker), counterstained with hematoxylin, and examined using an Olympus BX51 light microscope. An elongated roof plate (RP) and dorsal spinal cord malformation were observed in the *Wwox*$^{-/-}$ mouse embryo. Scale bars = 200 μm. **(b)** Decreased Ki-67-positive proliferating cells were detected in the neocortical subventricular zone (SVZ; upper panel) and cerebellum (arrows; lower panel) of *Wwox*$^{-/-}$ mouse embryo at E16.5. CP, cortical plate; IZ, intermediate zone; SVZ, subventricular zone; VZ, ventricular zone. Scale bars = 100 μm (upper) and 50 μm (lower). **(c)** The thickness of neocortex in the developing mouse brains at E16.5 was measured. Comparisons were made by one-way analysis of variance (ANOVA). *, $p < 0.05$.

Supplementary Figure 6

a

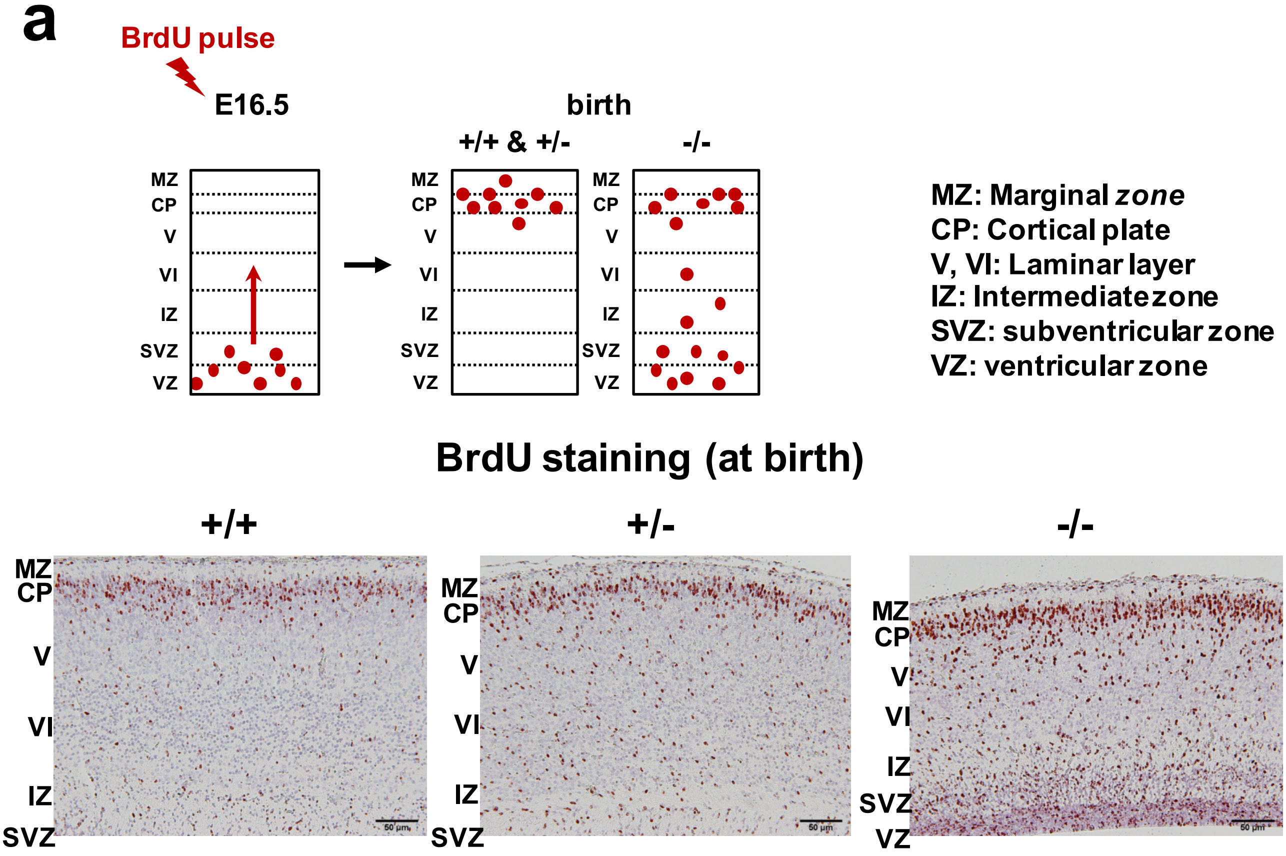

b

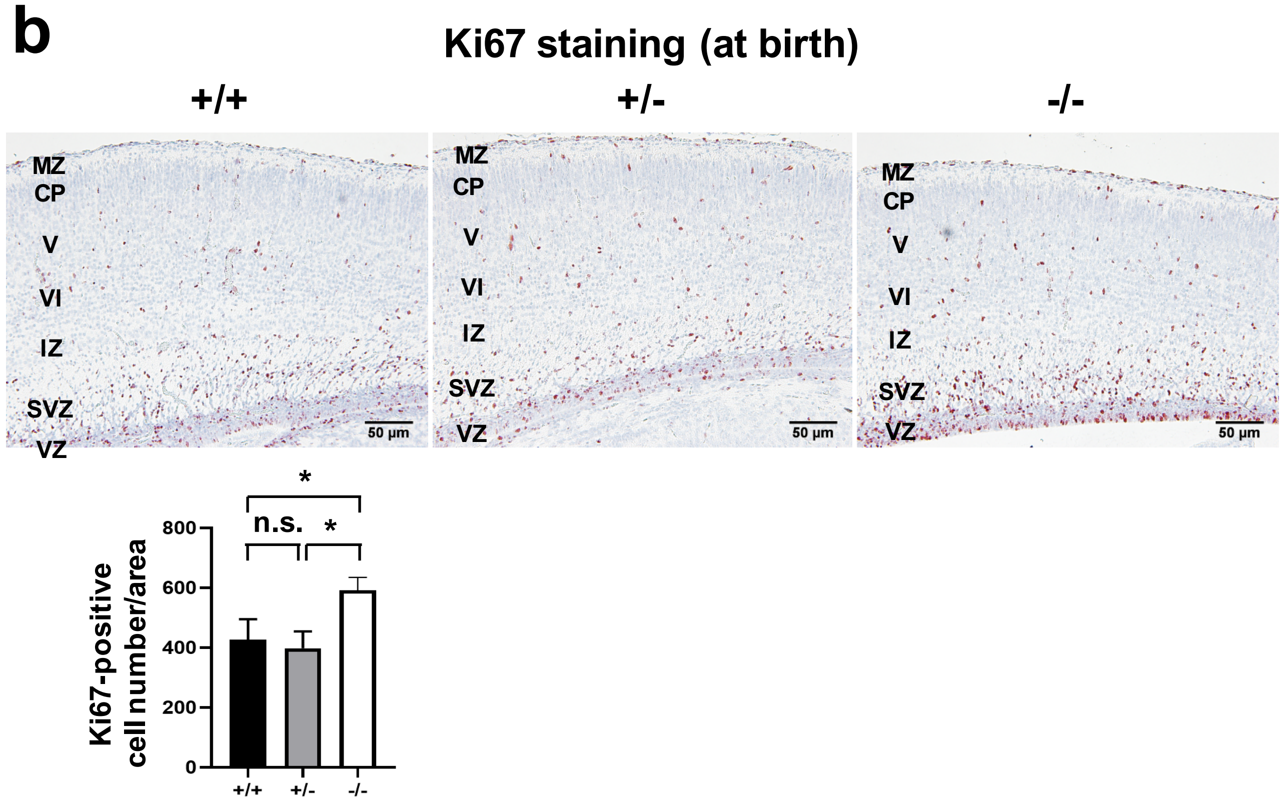

Supplementary Figure 6. *Wwox*$^{-/-}$ mouse neocortical neurons retain high proliferative activity after E16.5 and have poor mobility during development. **(a)** Following a pulse of bromodeoxyuridine (BrdU) to pregnant dams for labeling the actively proliferating cells in *Wwox*$^{+/+}$, *Wwox*$^{+/-}$ and *Wwox*$^{-/-}$ mouse embryos at E16.5, the brains collected from the pups at birth were fixed in 3.7% formaldehyde/PBS and embedded in paraffin. The neocortical tissue sections from these newborn mice were stained with an anti-BrdU antibody (GE Healthcare Life Sciences), followed by horseradish peroxidase (HRP)-conjugated anti-mouse IgG antibody staining and development with 3-amino-9-ethylcarbazole (AEC) substrate chromogen (Zymed). The tissue samples were counterstained with hematoxylin solution, mounted in aqueous mounting media, and examined using an Olympus BX51 light microscope. Scale bars = 50 μm. **(b)** The brain tissue sections from *Wwox*$^{+/+}$, *Wwox*$^{+/-}$ and *Wwox*$^{-/-}$ newborn mice were stained with an anti-Ki67 antibody (Dako) for detecting the actively proliferating cells. After HRP-conjugated secondary antibody staining, the samples were developed with AEC substrate chromogen, and examined using an Olympus BX51 light microscope. Scale bars = 50 μm. The representative images of three independent experiments are shown. The numbers of Ki67-positive cells in five representative subareas of *Wwox*$^{+/+}$, *Wwox*$^{+/-}$ and *Wwox*$^{-/-}$ cerebral cortex tissue sections were quantified in the lower panel. The results are expressed as means ± SEM. n.s., non-significant. *$P < 0.05$.

Supplementary Figure 7

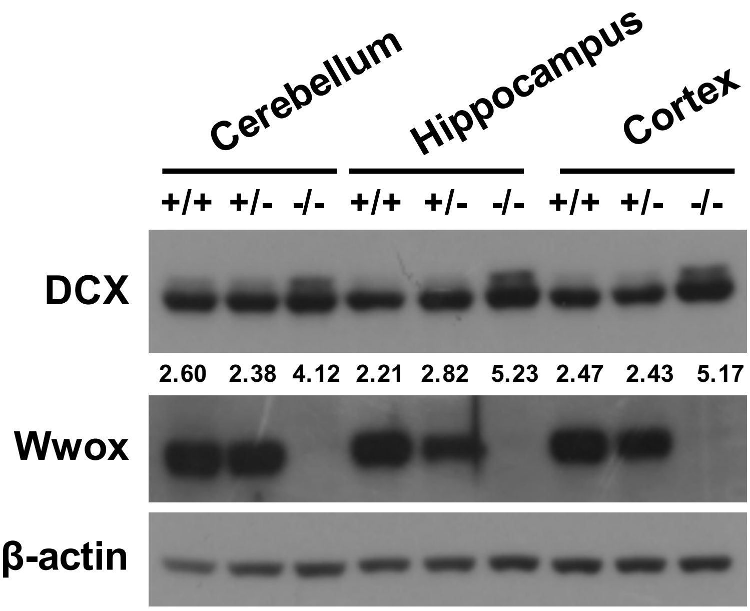

Supplementary Figure 7. *Wwox* loss leads to the increased DCX protein levels in mouse brain tissues at postnatal day 14. Cerebellum, hippocampus and cerebral cortex protein samples from three genotypes of mice at postnatal day 14 were examined for the expression levels of an early neuronal differentiation marker DCX by western blotting. β-actin was used as an internal control. Quantitative densitometry of the immunoblots was performed and the numbers depict the ratio of DCX to β-actin protein level in the brain tissues. The representative results of six independent experiments are shown.

Supplementary Figure 8

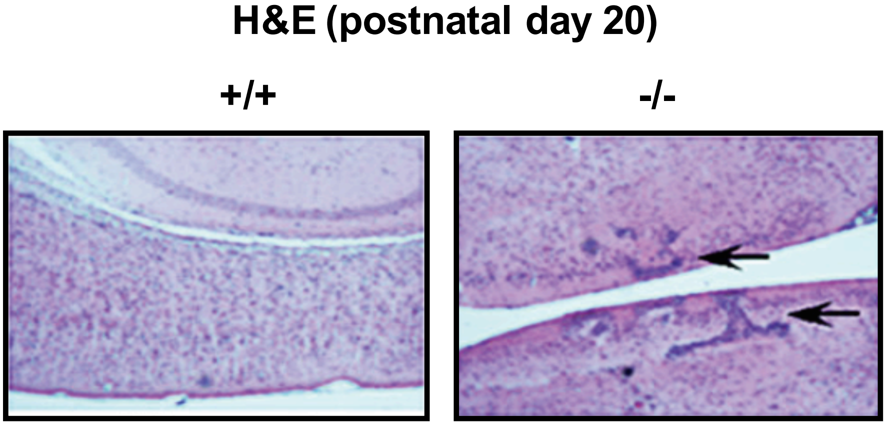

Supplementary Figure 8. Neuronal heterotopia (arrows) can be observed in the cortex of *Wwox* knockout mouse brain. The brains collected from *Wwox*$^{+/+}$ and *Wwox*$^{-/-}$ mice at postnatal day 20 were fixed in 3.7% formaldehyde/PBS and embedded in paraffin. Five-μm tissue sections were prepared, stained with hematoxylin and eosin (H&E) staining solutions, and examined using an Olympus BX51 light microscope.

Supplementary Figure 9

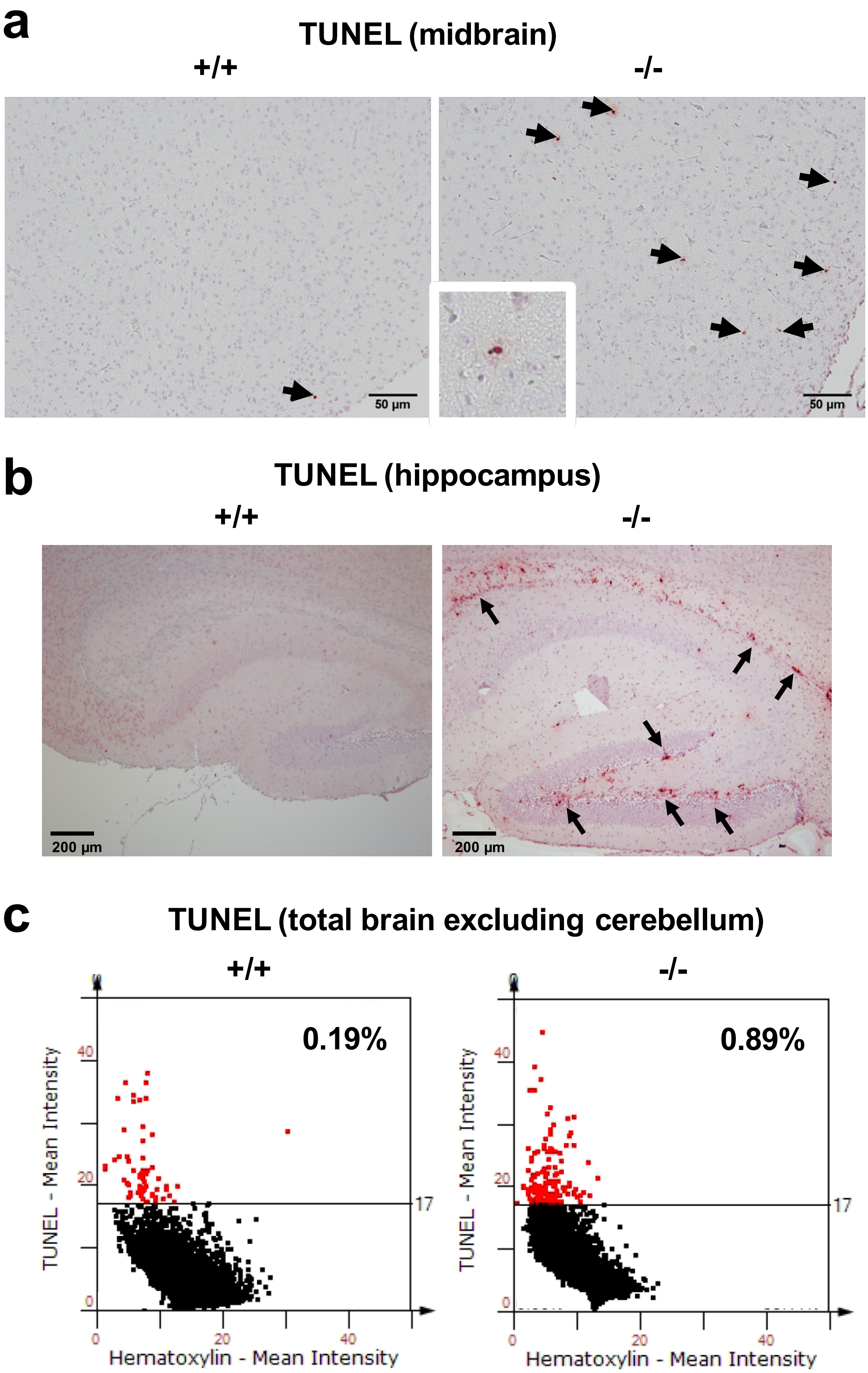

Supplementary Figure 9. Increased neuronal apoptosis is detected in *Wwox* knockout mouse brain. **(a,b)** Terminal deoxynucleotidyl transferase dUTP nick end labeling (TUNEL) assay was performed using sagittal brain sections from *Wwox*$^{+/+}$ and *Wwox*$^{-/-}$ mice at postnatal day 20. The tissue samples were counterstained with hematoxylin solution, mounted in aqueous mounting media, and examined using an Olympus BX51 light microscope. Increased TUNEL-positive apoptotic cells (arrows) were observed in *Wwox*$^{-/-}$ mouse midbrain **(a)** and hippocampus **(b)**. The insert image at the right panel is an enlargement of a TUNEL-positive cell. Scale bars represent 50 μm **(a)** and 200 μm **(b)**. **(c)** Sagittal brain sections from *Wwox*$^{+/+}$ and *Wwox*$^{-/-}$ mice at postnatal day 20 were used for TUNEL assay. After counterstaining with hematoxylin (X-axis), the brain sections were scanned and the increased intensity of TUNEL signal (Y-axis) in *Wwox*$^{-/-}$ mouse brain was analyzed using a TissueFAXS Imaging System. Compared with the wild-type, an increased percentage of apoptotic neurons was detected in total *Wwox*$^{-/-}$ mouse brain excluding the cerebellum.
